# AMBRA1 drives gastric cancer progression through regulation of tumor plasticity

**DOI:** 10.3389/fimmu.2024.1494364

**Published:** 2024-12-10

**Authors:** Liuqi Ye, Danlei Lin, Wen Zhang, Shiji Chen, Yumiao Zhen, Sara Akkouche, Xiaoxu Liang, Cheong-Meng Chong, Hai-Jing Zhong

**Affiliations:** ^1^ State Key Laboratory of Bioactive Molecules and Druggability Assessment, International Cooperative Laboratory of Traditional Chinese Medicine Modernization and Innovative Drug Development of Chinese Ministry of Education (MOE) of China, School of Pharmacy, Jinan University, Guangzhou, China; ^2^ School of Arts and Science, Guangzhou Maritime University, Guangzhou, China; ^3^ State Key Laboratory of Quality Research in Chinese Medicine, Institute of Chinese Medical Sciences, University of Macau, Macao, Macao SAR, China; ^4^ Key Laboratory of Prevention, Diagnosis and Therapy of Upper Gastrointestinal Cancer of Zhejiang Province, Zhejiang Cancer Hospital, Hangzhou, China

**Keywords:** AMBRA1, cell cycle, senescence, gastric cancer, tumor plasticity

## Abstract

**Background:**

Stomach adenocarcinoma (STAD) is an aggressive malignancy characterized by high tumor plasticity and heterogeneity. This study investigates the role of Autophagy and Beclin 1 Regulator 1 (AMBRA1) in regulating tumor plasticity in STAD progression.

**Methods:**

Combined with clinical data, the pan-cancer analysis of AMBRA1 was performed to analyze the role of AMBRA1 in STAD. Western blot, Flow Cytometry (FCM) assay, trans-well assay, wound healing assay, MTT, Reactive Oxygen Species (ROS) assay, Reverse Transcription Quantitative Polymerase Chain Reaction (RT-qPCR) and staining were performed to study the effects of AMBRA1 in AGS human gastric cancer cells. An AGS gastric cancer xenograft model was constructed to further verify the role of AMBRA1 in the development of STAD.

**Results:**

AMBRA1 overexpression correlated with poor overall survival in STAD and was positively associated with T cell CD4+ infiltration. High AMBRA1 expression also indicated worse prognosis in patients with high cancer-associated fibroblast infiltration. AMBRA1 depletion suppressed STAD cell proliferation, migration, and invasion *in vitro*. Mechanistically, AMBRA1 knockdown induced G1/S cell cycle arrest and triggered cellular senescence through epigenetic alterations, including changes in H3K9me3 levels. AMBRA1 inhibition also sensitized STAD cells to chemotherapeutic agents. *In vivo* studies confirmed the tumor-suppressive effects of AMBRA1 loss, resulting in reduced tumor growth and increased cellular senescence.

**Conclusions:**

Our findings uncover an oncogenic role for AMBRA1 in STAD. Targeting AMBRA1 may induce tumor cell senescence, apoptosis, and potentiate anti-tumor immunity, providing a rationale for developing AMBRA1-targeted precision therapies to improve clinical outcomes in STAD patients.

## 1 Introduction

Gastric cancer ranks among the deadliest malignancies globally, presenting significant challenges in treatment due to limited therapeutic options (1). A significant hurdle in its treatment is tumor plasticity, which leads to tumor heterogeneity, resistance to therapy, and malignant progression (2, 3). Recent studies indicated that cancer cell differentiation encompasses a complex array of stages, influenced by epigenetic alterations and dynamic responses to the tumor microenvironment (4–6). In this context, understanding the molecular mechanisms that regulate cellular plasticity is crucial for developing more effective treatment strategies.

One protein that has emerged as a potential key player in cellular plasticity is AMBRA1. AMBRA1 regulates various cellular processes, including autophagy, cell cycle progression, and epithelial-mesenchymal transition (EMT), underscoring its potential importance in cellular plasticity (7, 8). Given AMBRA1’s diverse cellular functions, its role in cancer development and progression has become a subject of increasing interest (9–11). Previous research has shown that AMBRA1 can function as both a tumor suppressor and an oncogene, depending on the cancer type (9, 12, 13). For instance, while AMBRA1 acts as a tumor suppressor in melanoma, it is overexpressed in several malignancies, including pancreatic ductal adenocarcinoma (PDAC) (14), cholangiocarcinoma (CHOL) (15) and STAD (16).

In the context of gastric cancer, extensive research on the role of AMBRA1 is still lacking. Our preliminary bioinformatics analysis revealed that AMBRA1 is significantly overexpressed in STAD tissues compared to normal gastric tissues and correlates with poor overall survival rates. Furthermore, high AMBRA1 expression is associated with increased immune infiltration, particularly T cell CD4+ levels, indicating its potential role in modulating the tumor microenvironment.

Given these insights, our study aims to elucidate the role of AMBRA1 in regulating tumor plasticity in gastric cancer. By investigating the functional implications of AMBRA1 expression in STAD, we found that AMBRA1 is highly expressed in STAD and poor overall survival and prognosis in STAD patients. Further investigation revealed that the deletion of AMBRA1 prevented the growth of gastric cancer cells and encouraged a reduction in cyclinD1 levels in human gastric cancer AGS cells. In addition, AMBRA1 deficiency promoted DNA damage, increased oxidative stress, and shortened telomere length, resulting in gastric cancer cell senescence, apoptosis, and ultimately, cell death. Furthermore, the increased drug sensitivity observed in AMBRA1 deficient cells suggests potential therapeutic benefits of AMBRA1 suppression in the treatment of gastric cancer. Loss of AMBRA1 aids in inhibiting cell invasion and metastasis of gastric adenocarcinoma. This research aims to elucidate the molecular mechanisms underlying AMBRA1’s involvement in gastric cancer progression.

## 2 Methods

### 2.1 AMBRA1 expression data analysis

The expression of AMBRA1 was analyzed using the TIMER2.0 database (17) with TCGA pan-cancer data. RNA-seq gene expression data, along with phenotypes and somatic mutations from TCGA-STAD tumors, were obtained from UCSC Xena (https://xena.ucsc.edu/) for further analysis (18). AMBRA1-related data were specifically extracted from these datasets using Perl (V5.32.1) for correlation analysis. AMBRA1 expression levels were examined across both normal tissues and 33 different tumor types. Additionally, TIMER 2.0 allows users to analyze immune infiltration by selecting CD4+ T cells for correlation analysis with AMBRA1 expression, generating scatter plots of gene expression correlations in STAD. The study also analyzed the relationship between AMBRA1 expression and patient prognosis using clinical data from UCSC Xena for Cox regression analysis. The cBioPortal tool was utilized to examine alterations in AMBRA1 gene characteristics, including the frequency, mutation types, and characteristics (19). Additionally, the R package was used to perform tumor mutational burden (TMB) and microsatellite instability (MSI) calculations to determine if AMBRA1-related alterations impact survival differences.

### 2.2 Cell culture and treatment

All cell lines were obtained from iCELL. AGS was maintained in F12 medium, while MKN-1 and GES-1 were maintained in RPMI-1640, supplemented with 10% FBS (Gibco).

### 2.3 Generation of Knockout (KO) cell lines via CRISPR-Cas9 technology

Heterogeneous KO cell populations were established using the lentiCRISPR v2 system. To facilitate the cloning of sgRNA oligonucleotides, a silent mutation was introduced into the ampicillin resistance open reading frame (ORF) that specifically targeted a BsmBI restriction site, preventing unwanted fragmentation of the vector during the cloning process. The sgAMBRA1 sequence (#4) was 5’-GAA CCA TAA TAT CTA TAT TA -3’ and was cloned as described previously (20). The AMBRA1 overexpressing plasmid was obtained from GeneCopoeia. (Catalog #EX-I0904-Lv242-B). The sgRNA-containing plasmid was transfected into cells at a concentration of 0.5 μg/mL, starting at the time of cell seeding, and this process was maintained for 72 hours. Subsequently, stable clones were selected based on puromycin resistance. KO confirmation was achieved through western blot analysis, followed by phenotypic assessment to evaluate the effects of AMBRA1 deficiency.

### 2.4 siRNAs and sgRNAs transfection methods

Unless otherwise noted, cells were transfected with siRNA at the time of seeding at a concentration of 20 nM for 48 hours. The following is a list of the specially created siRNA sequences for AMBRA1: siAMBRA1 (#1): 5’- GGC CUA UGG UAC UAA CAA ATT-3’, siAMBRA1 (#2): 5’- AGA ACU GCA AGA UCU ACA ATT -3’, siAMBRA1 (#3): 5’- GGC CCU AUG GUA CUA ACA ATT-3’. siAMBRA1 refers to siAMBRA1 #1 unless otherwise noted. Following the manufacturer’s instructions, LipofectamineTM 3000 Transfection Reagent (ThermoFisher Scientific, MA, USA, Catalog #11668-019) was used for all transfections.

### 2.5 Antibodies

Rabbit antibodies, including anti-AMBRA1 (Catalog #38182), anti-p27 (Catalog #41299), anti-p38 (Catalog #33149), anti-FoxO3A (Catalog #40937), anti-CUL4A (Catalog #38477), anti-CyclinD1 (Catalog #39315), anti-DDB1 (Catalog #38485), anti-Histone H2AX (Catalog #54667), anti-Phospho-Histone H2A.X (S139) (Catalog #13343), anti-Histone H3K9me3 (Catalog #HW029), anti-FoxO3A (Catalog #40937), anti-p53 (Catalog #HW109), anti-Caspase-3 (Catalog #9662), anti-Cleaved Caspase-3 (Catalog #9661), and anti-Phospho-Thr286 CyclinD1 (Catalog #11968), were procured from Signalway Antibody LLC (SAB). The anti-AMBRA1 antibody was used at a dilution of 1:1420 for Western blotting, while the other antibodies were used at a dilution of 1:1000. Mouse antibodies for actin (Catalog #FD0060), GAPDH (Catalog #FD0063), and tubulin (Catalog #FD0064) were obtained from Fdbio and applied at a dilution of 1:5000 for Western blotting.

### 2.6 MTT assay

AGS cells in their logarithmic growth phase were seeded into 96-well cell culture plates at a density of 5×10^3^ cells per well, with each well containing a volume of 100 μL. After allowing the cells to adhere, the culture solutions containing various concentrations of drugs were added to the experimental groups. Blank control wells were also set up. The plates were incubated at 37°C for 24 hours. Following incubation, the culture medium was removed, and 1 mg/mL MTT solution was added to each well. The cells were cultured for an additional 4 hours before adding DMSO to solubilize the formazan crystals. Absorbance was then measured at 570 nm.

### 2.7 Clonogenic assay

In a 6-well cell culture plate, 500 cells were seeded per well and cultured until positive clones were observed under a microscope. Following a 14-day incubation period, the cells were fixed with methanol for 30 minutes and stained with 0.1% crystal violet for 3 minutes. Excess crystal violet was removed by rinsing with PBS. Photographs were then taken using a camera, and the images were analyzed using Image J software to quantify the proliferating cells by calculating the stained area or colony number.

### 2.8 Wound healing assay

After 48 hours of transfection, AGS cells were seeded into 6-well plates and incubated for 24 hours, then manually scratched with scratches measuring 4 mm each. The cells were washed twice with PBS, and the basal medium containing drugs was added. Wound closure was monitored over time at specified intervals. Migrating cells were imaged using Zen 2.3 software on the Zeiss Cell Discoverer 7 Live Cell Workstation. The percentage of wound healing at the specified time points was calculated using Image J version 1.52, relative to the original area (T_0_).

### 2.9 Transwell assay

The Matrigel (Yeason, China) was taken out from -20°C and allowed to thaw overnight at 4°C. It was then diluted with F12 medium at a 1:8 ratio. In the invasion experiment, 60 μL of the diluted Matrigel were added to the upper chamber of the NuncTM polycarbonate cell culture inserts. The insert was incubated at 37°C to form a matrix membrane. After incubation, F12 medium was added to hydrate for 1 h before the cells were seeded. This step was not performed for the migration experiment. After transfection, AGS cells were cultivated for 48 hours and then seeded into 6-well cell culture plates. The cell monolayer was manually scratched every 4 mm after a 24-hour incubation period. Following two PBS washes, the cells were given a drug-containing basal media. At predefined intervals, wound closure was noted. Cells were photographed during migration using the Zeiss CellDiscoverer 7 Live Cell Workstation and Zen 2.3 software. Data are shown as mean counts.

### 2.10 TUNEL staining assay

AGS cells were seeded into confocal dishes at a density of 1×10^5^ cells per well. Following adherence, experimental groups were treated with culture solutions containing various drug concentrations. After being fixed for 20 minutes with 4% formalin, the apoptotic AGS cells were permeabilized for 5 minutes with 0.3% Triton X-100 (w/v) in PBS. A One Step TUNEL Apoptosis Assay Kit (Red Fluorescence) (Beyotime, China) was used to measure apoptosis, and DAPI (1µg/mL) was used to stain the nuclei. Added 50 μL of TUNEL detection solution and incubated at 37°C in the dark for 60 minutes, followed by washing twice with PBS. The specimen was then mounted with an anti-fade mounting medium and observed under a fluorescence microscope, with the Cy3 excitation wavelength set at 550 nm and the emission wavelength at 570 nm.

### 2.11 Annexin V/PI staining

After centrifuging AGS cells for 5 minutes and discarding the supernatant, the cells were recovered and given a single PBS wash. The cells were gently resuspended and counted. Subsequently, 1×10^5^ cells were resuspended and subjected to another 5-minute centrifugation, after which the supernatant was discarded. Following an additional PBS wash, the cells were centrifuged again, and the supernatant was discarded. To resuspend the cells, 100 μL of diluted 1× Annexin V Binding Buffer was added. The cell suspension was then mixed with 2.5 μL of PI Reagent (1 µg/mL) and 2.5 μL of Annexin V-FITC Reagent. The mixture was gently vortexed to ensure complete mixing and incubated at room temperature in the dark for 20 minutes. After the incubation period, the sample was thoroughly mixed with 400 μL of diluted 1× Annexin V Binding Buffer. The material was then immediately processed using FlowJo v10.8.1 software and prepared for detection on the BD Bioscience FACSCanto cytometer.

### 2.12 SA-β-gal assay

The AGS cells were seeded in a six-well plate at a density of 4×10^5^ cells per well. After reaching the desired confluence, The cells were initially fixed using a 2% formaldehyde and 0.2% glutaraldehyde solution for 15 minutes. This was followed by a PBS wash. After that, they were incubated with SA-β-Gal staining solution (Beyotime, Catalog #C0602) for a whole night at 37**°**C. The cells were inspected under a microscope following two PBS rinses. Counting at least 500 cells allowed us to determine the percentage of SA-β-gal-positive cells.

### 2.13 ROS detection

ROS measurement was performed in 96-well cell culture plates by seeding cells at a density of 8×10^3^ cells/well. During the detection period, cells underwent two PBS washes before a final concentration of 5 µM of carboxy-DCFDA dye was introduced in serum-free media. The plates were incubated for 20 minutes at 37°C in order to remove the dye. Nuclear staining dye was applied, and the samples were examined right away using a high-content imaging device (Fluorescence Microplate Reader, TECAN).

### 2.14 Quantitative Real-Time Polymerase Chain Reaction

Following treatment, total cellular RNA was extracted from AGS cells using the RNA simple Total RNA Kit (TIANGEN BIOTECH, China) as per the manufacturer’s instructions. The extracted RNA was then reverse transcribed into cDNA using the RT Master Mix (TIANGEN BIOTECH, China). The quantity and purity of the RNA were assessed using a NanoDrop 2000 (Thermo Fisher Scientific).The primer sequences are Telomere F: 5′- CGG TTT GTT TGG GTT TGG GTT TGG GTT TGG GTT TGG GTT -3′. R: 5′- GGC TTG CCT TAC CCT TAC CCT TAC CCT TAC CCT TAC CCT -3′. 36B4 F: 5′- CAG CAA GTG GGA AGG TGT AAT CC -3′. R: 5′- CCC ATT CTA TCA TCA ACG GGT ACA A -3′. Quantitative PCR (qPCR) was conducted using a qPCR SYBR Green Master Mix (Yeason, China). The relative expression levels of the target genes were determined using the comparative (2-^△△Ct^) method.

### 2.15 Pathway interaction database and enrichment analysis

The pathway network of human AMBRA1 was explored using the pathway interaction database (http://www.ndexbio.org/) curated by STRING (21). Visualization of pathway analysis was conducted using Cytoscape (3.9.0). The top 100 related genes were selected for gene ontology (GO) enrichment analysis using Gene Set Enrichment Analysis (GSEA) (4.0.3). Data processing via the R packages “clusterProfiler”, “pathview”.

### 2.16 *In vivo* model

5×10^6^ KO of AMBRA1 AGS gastric cancer cells were resuspended in 100 μL of sterile PBS and mixed at a 1:1 ratio with an equal volume of Ceturegel matrix gel (Yeason, China). The mixture was injected subcutaneously into the flank area of 6-7 weeks old male BALB/c-nude mice, with six mice per experimental group. When the tumor volume reached or exceeded 1×10^3^ mm³, or at the predetermined endpoint of 21 days, the mice were euthanized to terminate the experiment. Subsequently, the subcutaneous tumors were removed, weighed, and photographed.

### 2.17 Hematoxylin & eosin (H&E) and immunohistochemistry (IHC) staining

Tissue sections were subjected to normal histology methods, which involved deparaffinization using xylene and staining with hematoxylin and eosin (Nanjing Jiancheng, China). For IHC staining, after the sections were deparaffinized and rehydrated, the specimens were incubated using a high-pressure technique in 1 mM EDTA buffer (pH 8.0) to perform antigen retrieval for IHC labeling. Following that, the tissue sections were treated with primary antibodies anti-AMBRA1 (Affinity, cat#DF6228), anti-Ki67 (Beyotime, cat#AF1738), anti-H3K9me3 (SAB, Catalog #HW029), and anti-γ-H2AX (SAB, Catalog #53210) at a dilution ratio1:50 for an entire night at 4**°**C. Target proteins were identified by using a DAB solution (GT-BIO, China), which conjugated the peroxidase enzyme to produce a brown precipitate.

### 2.18 Image processing and analysis

GraphPad 7.0 was used to analyze all of the high-content imaging system’s images, and Image J was employed to quantify the western blot signals.

### 2.19 Statistical analysis

All gene expression data were normalized, and statistical significance was determined at *p <* 0.05. Statistical analyses, including one-way ANOVA and two-way ANOVA, were performed using Perl (V5.32.1) and R software (version 4.1.3). In the figures, one-way ANOVA was applied to compare two or more independent groups, while two-way ANOVA was conducted to estimate changes in quantitative variables. The following notation is used to indicate the level of significance: *p < 0.05, **p < 0.01, ***p < 0.001, ****p < 0.0001.

## 3 Result

### 3.1 High expression of AMBRA1 showed a worse prognosis and clinicopathological characteristics of STAD

**Figure 1:**
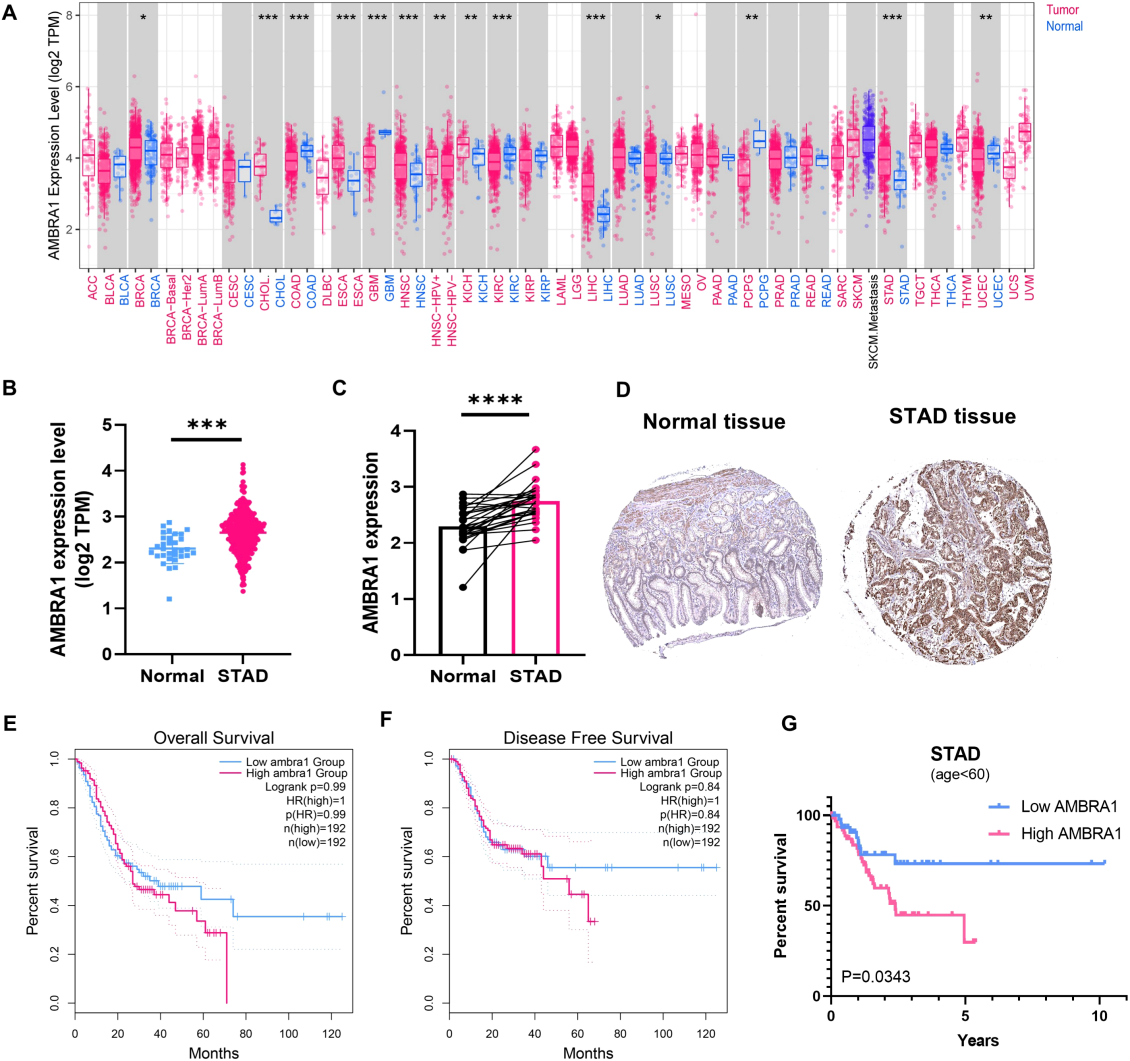
AMBRA1 is highly expressed in STAD and closely associated with worse prognosis. **(A)** AMBRA1 expression in different types of cancers (versus non-tumor tissues) in the TCGA dataset. The samples’ log2 (TPM) values for AMBRA1 expression are shown on the Y-axis. AMRBA1 pan-cancer expression study in tumor and normal tissues using TIMER. **(B)** The levels of AMBRA1 between tumor and normal tissues of STAD. **(C)** AMBRA1 pairwise difference analysis in normal and tumor TCGA-STAD samples. **(D)** The IHC image from the Human Protein Atlas shows a visual representation of AMBRA1 protein expression in normal tissue and STAD tissue. **(E, F)** Kaplan-Meier analysis of the association between AMBRA1 expression with overall survival and disease-free survival rates. **(G)** Kaplan-Meier analysis of the association between AMBRA1 expression with overall survival under the age of 60. *p < 0.05; **p < 0.01; ***p < 0.001; ****p < 0.0001.

Analysis of data from the UCSC XENA database, which incorporates information from The Cancer Genome Atlas Program (TCGA), Genotype-Tissue Expression (GTEx), and etc., revealed significant upregulation of AMBRA1 expression in seven distinct solid tumors, including cholangiocarcinoma (CHOL), breast cancer, esophageal carcinoma (ESCA), kidney chromophobe, liver hepatocellular carcinoma, head and neck squamous cell carcinoma, and STAD (**Figures 1A, B**). Additionally, the pairwise difference analysis revealed that AMBRA1 levels were abnormally high and significantly elevated in STAD (**Figure 1C**). Immunohistochemistry (IHC) studies further corroborated these findings, demonstrating elevated AMBRA1 protein levels in STAD tissues when compared to normal tissues (**Figure 1D**). Moreover, patients exhibiting high AMBRA1 expression (using a 1.5-fold cut-off) were found to have worse overall survival (p(HR)=0.99) and disease-free survival rates (p(HR)=0.84) (**Figures 1E, F**). STAD patients under 60 years of age with high AMBRA1 expression had poorer prognosis (**Figure 1G**, *p*=0.0343). Analysis of TCGA data showed that the highest frequency of AMBRA1 genetic alterations occurred in uterine corpus endometrial carcinoma, melanoma, and STAD, with missense mutations being the primary alteration type (**Supplementary Figure S1**). TIMER 2.0 immune infiltration analysis further identified that high AMBRA1 expression shows a positive correlation with T cell CD4+ infiltration levels (*p*=6.42e-06), suggesting a role in immune regulation (**Supplementary Figure S1C**). Additionally, AMBRA1 mutations lead to a significant reduction in T cell CD4+ memory cell infiltration (*p*=0.024), potentially weakening the immune response to tumors (**Supplementary Figure S1D**). Survival analysis indicates that patients with high AMBRA1 expression and high cancer-associated fibroblast infiltration have poorer survival rates (*p*=0.0196, **Supplementary Figure S1E**). These suggest that AMBRA1 gene expression is significantly associated with immune infiltration and patient prognosis.

### 3.2 AMBRA1 deficiency suppresses AGS STAD cell proliferation, migration and invasion

**Figure 2:**
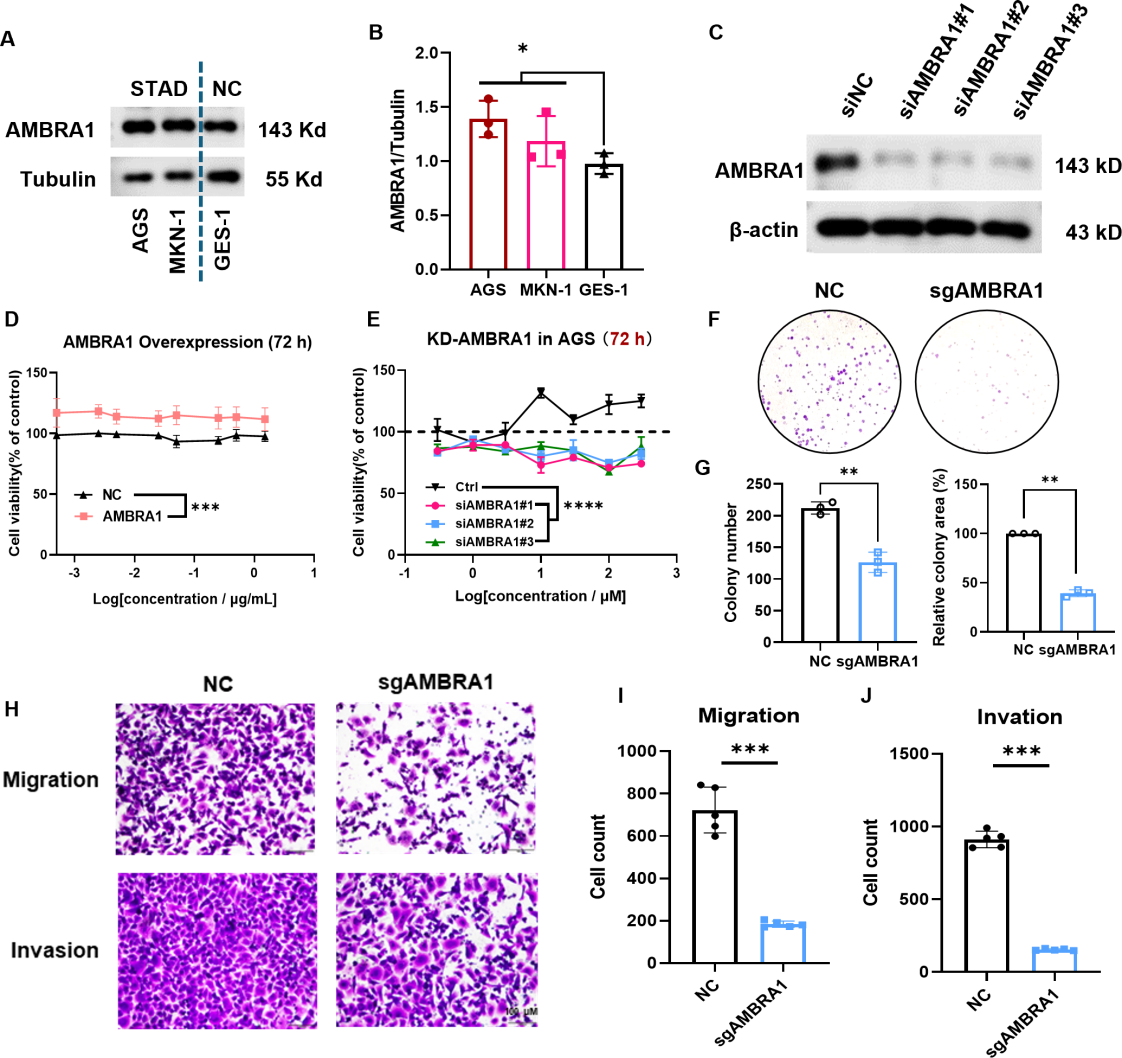
AMBRA1 is overexpressed in STAD cells and highly closed to gastric cancer cell growth, migration and invasion. **(A, B)** AMBRA1 was significantly overexpressed in the STAD cell lines AGS and MKN-1 compared to the normal human gastric epithelial cell line GES-1. **(C)** Western blotting analysis of the AMBRA1 levels after the treatment of control siRNA and siAMBRA1#1, siAMBRA1#2 and siAMBRA1#3. **(D)** AGS cells were transfected with control plasmid and AMBRA1 overexpressing plasmid for 72 h, and cell viability was measured using MTT assay. **(E)** AGS cells were transfected with control siRNA and siAMBRA1s for 72 h, and cell viability was measured using MTT assay. **(F)** Representative images and **(G)** quantification of colony numbers and relative colony area of ctrl sgRNA group and AMBRA1 sgRNA group stained after 7 days (n = 3 for each group). **(H-J)** Representative images and quantitative results for the migration and invasion assays in AGS cells with normal plasmid or KO-AMBRA1 plasmid transfection. *p < 0.05; **p < 0.01; ***p < 0.001; ****p < 0.0001.

Based on our bioinformatics analysis revealing the significant upregulation of AMBRA1 in STAD and its correlation with poor prognosis (**Figures 1E–G**), we sought to validate these clinical observations and explore the functional role of AMBRA1 through a series of *in vitro* experiments using STAD cell lines. AGS and MKN-1 are two commonly used cell lines in experimental models for gastric cancer research. GES-1, a normal gastric epithelial cell line, serves as a non-tumorigenic control, providing a baseline for comparative studies. Western blotting results showed significantly higher AMBRA1 levels in STAD cells compared to normal cells (*p <* 0.05, **Figures 2A, B**), which was aligned with the clinical data. Additionally, we generated AMBRA1 knockdown (KD), KO and overexpression models in the AGS gastric cancer cell line (**Figure 2C**; **Supplementary Figure S2**). Based on the correlation of AMBRA1 expression with patient survival, we hypothesized that AMBRA1 plays a critical role in promoting gastric cancer cell growth and invasion. MTT and colony formation assays were applied to investigate the effect of AMBRA1 on the growth of STAD cells. The outcomes showed that AGS cells with overexpression of AMBRA1 had noticeably higher rates of cell viability (**Figure 2D**). On the other hand, AGS cell growth was significantly suppressed by AMBRA1 KD (**Figure 2E**). Moreover, AMBRA1 KD cells exhibited increased sensitivity to growth inhibition by the proteasome inhibitor MG132, protein synthesis inhibitor cycloheximide (CHX), and NEDD8 activating enzyme small molecule inhibitor MLN4924, compared to the control group (**Supplementary Figure S3**), suggesting that AMBRA1 inhibition enhanced the sensitivity of STAD cells to chemotherapeutic agents. AMBRA1 KD could significantly inhibit colony formation (**Figures 2F, G**; **Supplementary Figure S4**). All of these results point to the critical function that AMBRA1 plays in controlling the growth of STAD cells.

Cell motility and invasion are key indicators of cancer malignancy (22). To assess the influence of AMBRA1 on AGS STAD cell motility and invasiveness, we employed wound healing and transwell assays. The results revealed that both AMBRA1 KO and KD significantly reduced the migration and invasion capabilities of AGS cells (**Figures 2H–J**; **Supplementary Figure S5**). These findings indicate that AMBRA1 is essential for maintaining the migratory and invasive capabilities of STAD cells. AMBRA1 depletion in AGS gastric cancer cells led to significant changes in cell morphology and behavior. We observed reduced growth, migration, and invasion capabilities, indicating AMBRA1’s role in maintaining the plastic nature of AGS cells.

### 3.3 AMBRA1 deficiency triggered cell cycle arrest, apoptosis in gastric cancer

**Figure 3:**
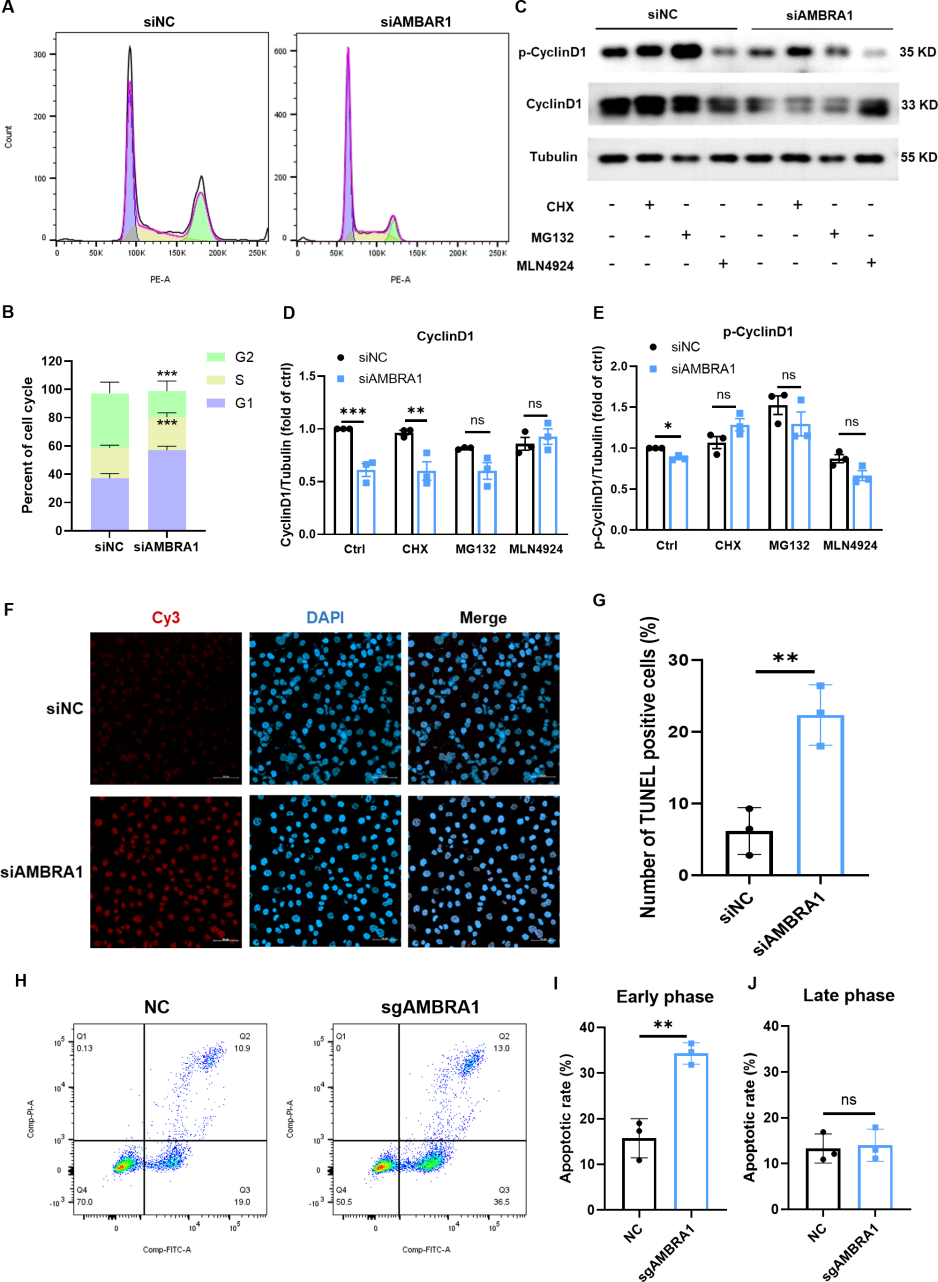
AMBRA1 KD induces cell cycle arrest and apoptosis in gastric cancer cells. **(A)** Representative flow cytometry histograms showing the cell cycle distribution in AGS cells transfected with control siRNA or siAMBRA1. Each curve indicates the distribution of cell counts across different phases of the cell cycle, allowing for a comparison between the effects of the negative control and AMBRA1 knockdown on cell cycle progression. Left (G1 Phase): The curve on the left corresponds to the G1 phase of the cell cycle, indicating cells that are in the initial growth phase. Middle (S Phase): The curve in the middle represents the S phase, where DNA replication occurs. Right (G2 Phase): The curve on the right indicates the G2 phase, which is the second growth phase before mitosis. **(B)** Quantification of the percentage of cells in G1, S, and G2 phases of the cell cycle in control and KD-AMBRA1 AGS cells. **(C)** The levels of AMBRA1, CyclinD1, p-CyclinD1 and Tubulin in AGS and KD-AMBRA1 cells under CHX (1 μM), MG132 (0.5 μM) and MLN4924 (5 μM)-treated for 24 hours were detected by western blotting. **(D, E)** Quantitative analysis of the level of Cyclin D1 in AGS cells and AMBRA1 KD AGS cells in Western blotting assay. **(F)** Representative fluorescence microscopy images showing TUNEL-positive apoptotic cells (light red dots) in AGS cells transfected with control siRNA or siAMBRA1. Nuclei are counterstained with DAPI (blue). **(G)** Quantification of the percentage of TUNEL-positive apoptotic cells in control and KD-AMBRA1 AGS cells. **(H)** Representative flow cytometry dot plots showing Annexin V-FITC (x-axis) and propidium iodide (PI, y-axis) staining in AGS cells transfected with control sgRNA or sgAMBRA1. **(I-J)** Quantification of the percentage of early/late apoptotic cells in control and KO-AMBRA1 AGS cells. *p < 0.05; **p < 0.01; ***p < 0.001.

The ultimate stage of cellular senescence is known as apoptosis, a highly controlled type of programmed cell death that is essential to preserving cellular homeostasis (9). Cell cycle and western blotting analysis revealed that AMBRA1-deficient AGS cells exhibited decreased cyclin D1 levels, increased DNA content in the G1 phase, decreased content in the G2 phase, and induced G1 phase arrest (**Figures 3A, B**). These findings align with the observed inhibitory effect on gastric cancer cell proliferation, suggesting that AMBRA1 deficiency induces cell cycle arrest. Notably, treatment with proteasome inhibitor MG132 or neddylation inhibitor MLN4924 significantly increased AMBRA1 and cyclin D1 levels in control (siNC) AGS cells (**Figures 3C–E**).

We further evaluated the impact of AMBRA1 on apoptosis using TUNEL and Annexin-V/Propidium iodide assays. TUNEL result showed that AMBRA1-silenced AGS cells showed a significant increase in apoptosis compared to control cells (*p <* 0.01, **Figures 3F, G**). After 48 hours of AMBRA1 KD, 22.4 ± 4.2% of cells were apoptotic, compared to 6.2 ± 3.3% in control cells (*p <* 0.01), representing a 3.6-fold increase in apoptosis rate. An Annexin-V/Propidium iodide flow cytometric study corroborated these findings, showing that AMBRA1 KO significantly increased the early apoptotic population to 34.3 ± 2.4% in AGS cells (*p <* 0.01, **Figures 3H–J**).

### 3.4 AMBRA1 deficiency leads to senescence of AGS STAD cells

**Figure 4:**
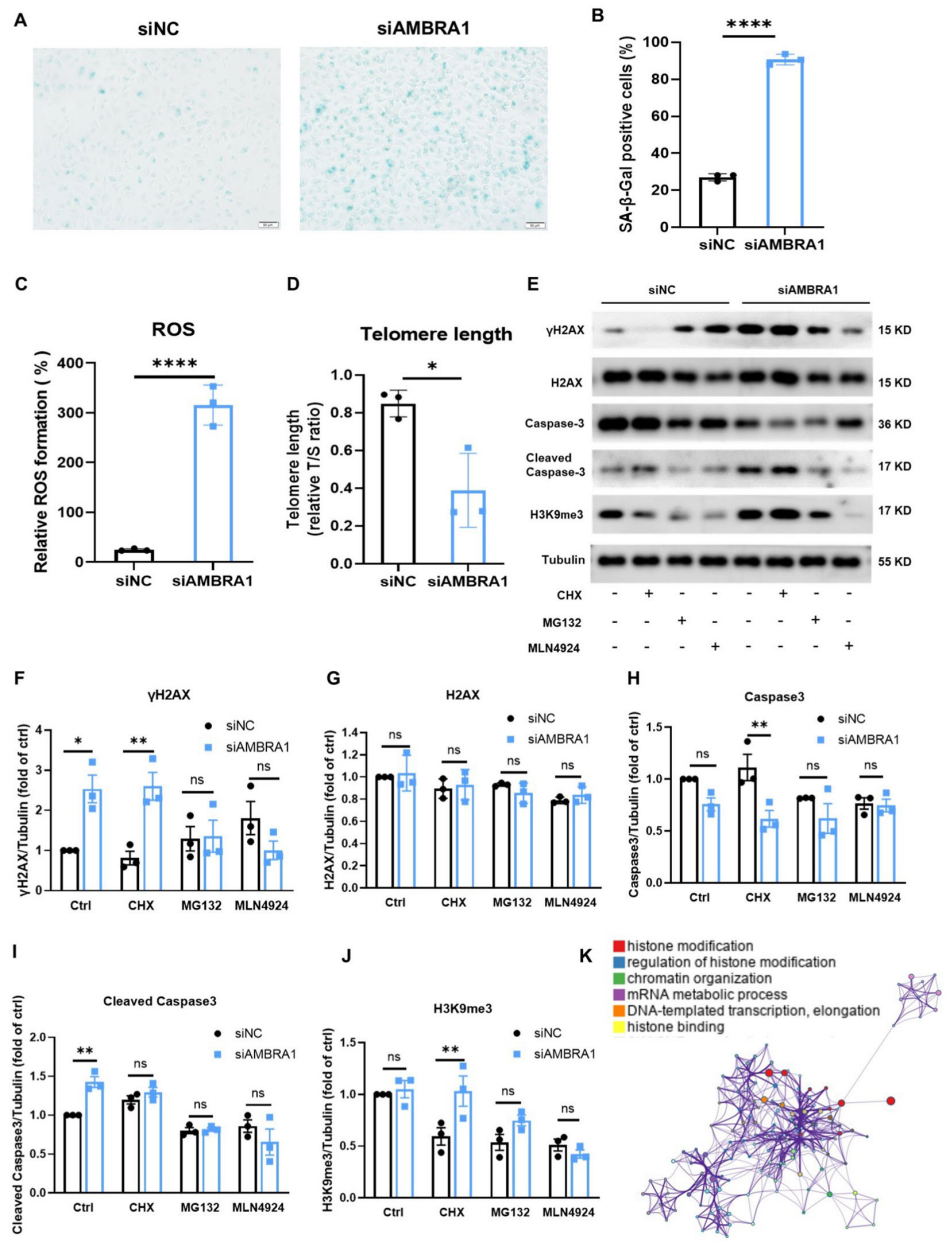
KD of AMBRA1 induced the senescence of AGS gastric cancer cells through oxidative stress, shortening telomere length, promoting H2AX phosphorylation. **(A)** Representative images and **(B)** quantification of SA-β-gal staining of AGS cells and KD-AMBRA1 AGS cells (n = 3 for each group). Scale bar: 50 μm. **(C)** Quantification of ROS levels detected by dihydroethidium (DHE) staining in AGS cells transfected with control siRNA or siAMBRA1. **(D)** Quantification of telomere length, in control and KD-AMBRA1 AGS cells. **(E)** The expressions of γH2AX, H2AX, Caspase3, cleaved caspase3, H3K9me3 and Tubulin in AGS and KD-AMBRA1 AGS cells under CHX (1 μM), MG132 (0.5 μM) and MLN4924 (5 μM)-treated for 24 hours were detected by western blotting. **(F–J)** Quantitative analysis of the level of Cyclin D1 in AGS cells and AMBRA1 KD AGS cells in Western blotting assay. **(K)** The GO enrichment analysis reveals that a significant number of the identified genes are associated with histone modification processes. *p < 0.05; **p < 0.01; ****p < 0.0001.

Interestingly, we observed the senescence-associated morphological changes in AMBRA1 deficient AGS cells. Cellular senescence is a state where cells undergo a prolonged and usually irreversible arrest in the cell cycle while acquiring different phenotypic changes. These changes include alterations in morphology, chromatin configuration, and a metabolic reprogramming (23). To confirm whether the cell senescence occur in the AMBRA1-KD AGS cells, we employed the senescence-associated beta-galactosidase (SA-β-Gal) staining assay, a widely recognized biomarker for senescent cells. Our results, as depicted in **Figures 4A, B**, revealed a striking increase in the proportion of SA-β-Gal positive cells following AMBRA1 KD (*p <* 0.0001). The observed enhancement of SA-β-Gal staining intensity and prevalence in AMBRA1-KD cells strongly suggests that AMBRA1 plays a critical role in regulating the senescence program.

Reactive oxygen species (ROS) buildup in metabolically active senescent cells causes oxidative DNA damage at telomeric G-rich repeats, leading to the development of what are known as telomere dysfunction-induced foci (TIFs), a component of the response to DNA damage (24, 25). We found that AMBRA1 KD resulted in a significant elevation of ROS levels in AGS cells (*p <* 0.0001, **Figure 4C**). This increase in oxidative stress is a hallmark of cellular senescence and can contribute to accelerated aging at the cellular level. Telomere length (TL) is another crucial factor in cellular senescence (26). To assess the impact of AMBRA1 deficiency on telomere integrity, we compared TL between the control (siNC) and AMBRA1 KD (siAMBRA1) groups. As illustrated in **Figure 4D**, the siAMBRA1 group exhibited significantly shorter telomeres compared to the siNC group (*p* = 0.019). This observation suggests that AMBRA1 deficiency may accelerate telomere attrition, potentially contributing to premature senescence.

DNA damage response (DDR) activities trigger the phosphorylation of histone H2AX (γH2AX) and the trimethylation of histone H3 at lysine 9 (H3K9me3). These modifications enhance the receptivity of chromatin to the binding and assembly of proteins and DNA repair machinery crucial for the DDR signaling cascade (27, 28). Our investigation into the effects of AMBRA1 deficiency on these DDR markers yielded significant results. As illustrated in **Figures 4E–J**, AMBRA1 depletion led to a marked increase in the levels of both γ-H2AX and H3K9me3 in AGS cells. AMBRA1 knockdown resulted in significant epigenetic alterations, including increased levels of H3K9me3, a marker associated with heterochromatin formation and gene silencing. This suggests AMBRA1’s involvement in epigenetic regulation of gene expression in STAD cells. Moreover, siAMBRA1 treatment resulted in elevated levels of cleaved caspase-3, a well-known apoptotic marker, in AGS cells. This finding suggests that AMBRA1 deficiency not only promotes cellular senescence but may also enhance apoptotic signaling. The GO enrichment analysis was conducted to explore the functional roles of the identified genes. The top 100 related genes were selected for analysis using GSEA (4.0.3). The result revealed a pronounced association between these genes and histone modification processes (**Figure 4K**). Histone modifications are crucial post-translational modifications that play a significant role in regulating gene expression by altering chromatin structure and accessibility. The enrichment of genes involved in histone modification suggests that these processes may be pivotal in cellular senescence and apoptosis.

**Figure 5:**
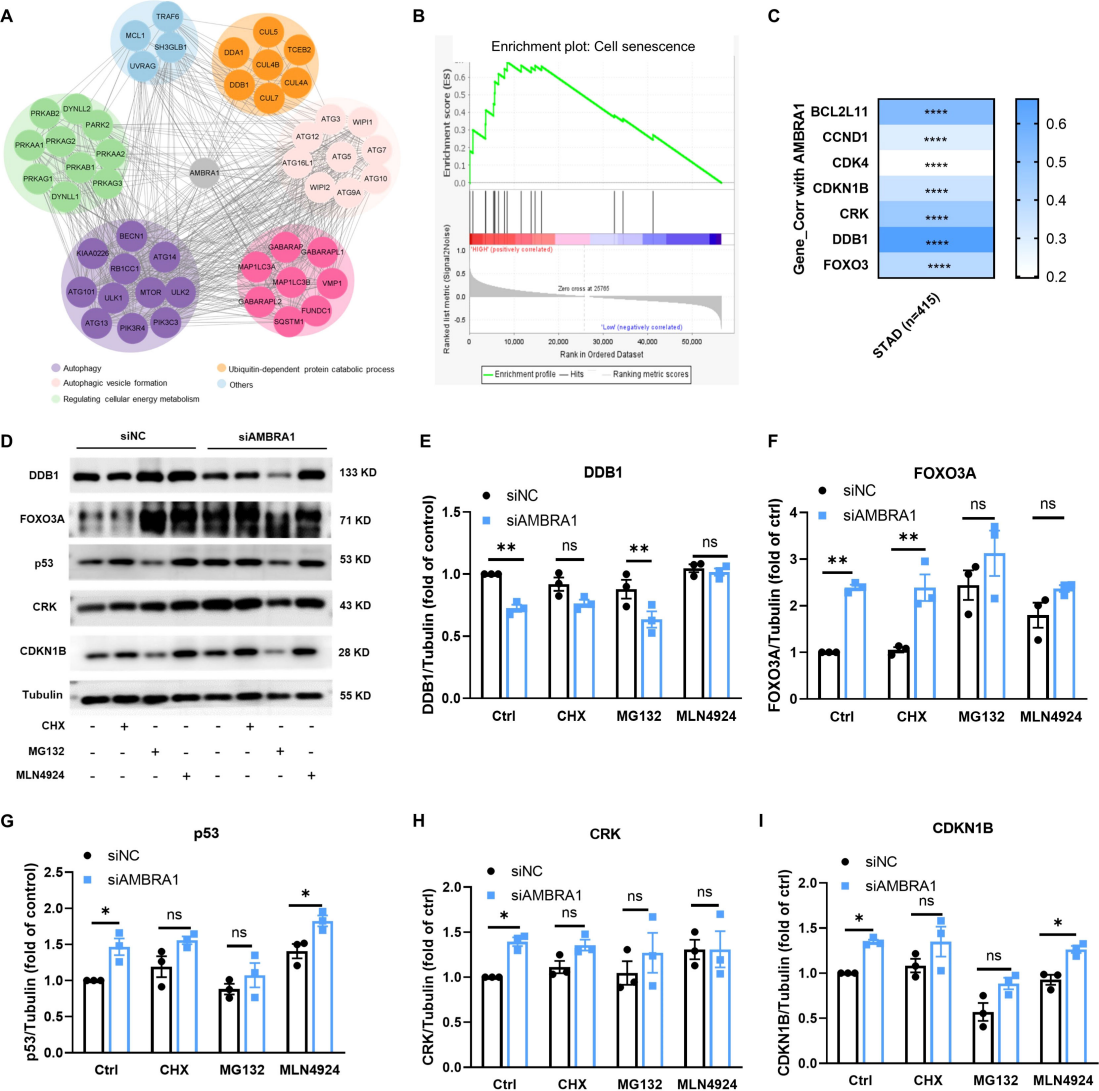
KD of AMBRA1 triggered the FoxO3A/p53/CRK/CDKN1B signaling pathway. **(A)** AMBRA1 pathway network derived from the Pathway Interaction Database which was curated by STRING. **(B)** GSEA of transcriptomic data from gastric cancer samples of The Cancer Genome Atlas (TCGA-STAD), senescence gene sets in AMBRA1_Low vs. AMBRA1_High subgroups (n = 16 for each subgroup). **(C)** Correlation between AMBRA1 and FOXO3, DDB1, CRK, CDKN1B, CDK4, CCND1, BCL2L11 in TCGA-STAD on TIMER2, with Spearman’s rho value representing the correlation degree. **D–I)** The expression of DDB1, FOXO3, DDB1, p53, CRK, CDKN1B and Tubulin in AGS and KD-AMBRA1 cells under CHX (1 μM), MG132 (0.5 μM) and MLN4924(5 μM)-treated for 24 hours were detected by western blotting. *p < 0.05; **p < 0.01; ****p < 0.0001.

To identify novel AMBRA1-binding proteins, we conducted a computational string search and constructed a STRING interaction network. The 50 closest AMBRA1 interacting proteins involved in autophagy, ubiquitin-dependent protein catabolic process, autophagic vesicle formation and regulation cellular energy metabolism, such as DDB1 (**Figure 5A**). To further identify the senescence gene sets in gastric cancer patients’ samples (n = 415), we conducted an enrichment analysis in TCGA-STAD dataset. Using the TCGA dataset, we conducted an enrichment analysis of ‘AMBRA1_Low vs. AMBRA1_High’ and discovered a significant activation of senescence-related genomes in STAD (refer to **Figure 5B**). Intriguingly, GSEA of all datasets revealed that the AMBRA1 subgroup was enriched with genes, including FOXO3, DDB1, CRK, CDKN1B, CDK4, CCND1, etc., associated with AGS cell senescence (**Figures 5B, C**). At the same time, senescence-related genes were used to generate the signature, and protein levels of expression were confirmed. The correlation between AMBRA1 and the cell senescence-related genes caused by the DDB1/FOXO3/CDKN1/CCND1 pathway in gastric cancer samples was explored, among which DDB1, FoxO3A, p53, CRK, and CDKN1B, were highly correlated as the enrichment result mentioned above (**Figures 5D–I**). Additionally, TCGA analysis further revealed a significant upregulation of DDB1, CUL4A, CCND1, and CDKN1 expression in STAD patients (**Supplementary Figure S6**). Taken together, our data proposed the increased ROS by siAMBRA1 exposure activated DDB1/FOXO3/CDKN1/CCND1 signaling, and the activated DDB1/FOXO3/CDKN1/CCND1 pathway was involved in the anti-proliferative effect of siAMBRA1 via inducing senescence in AGS cells.

**Figure 6:**
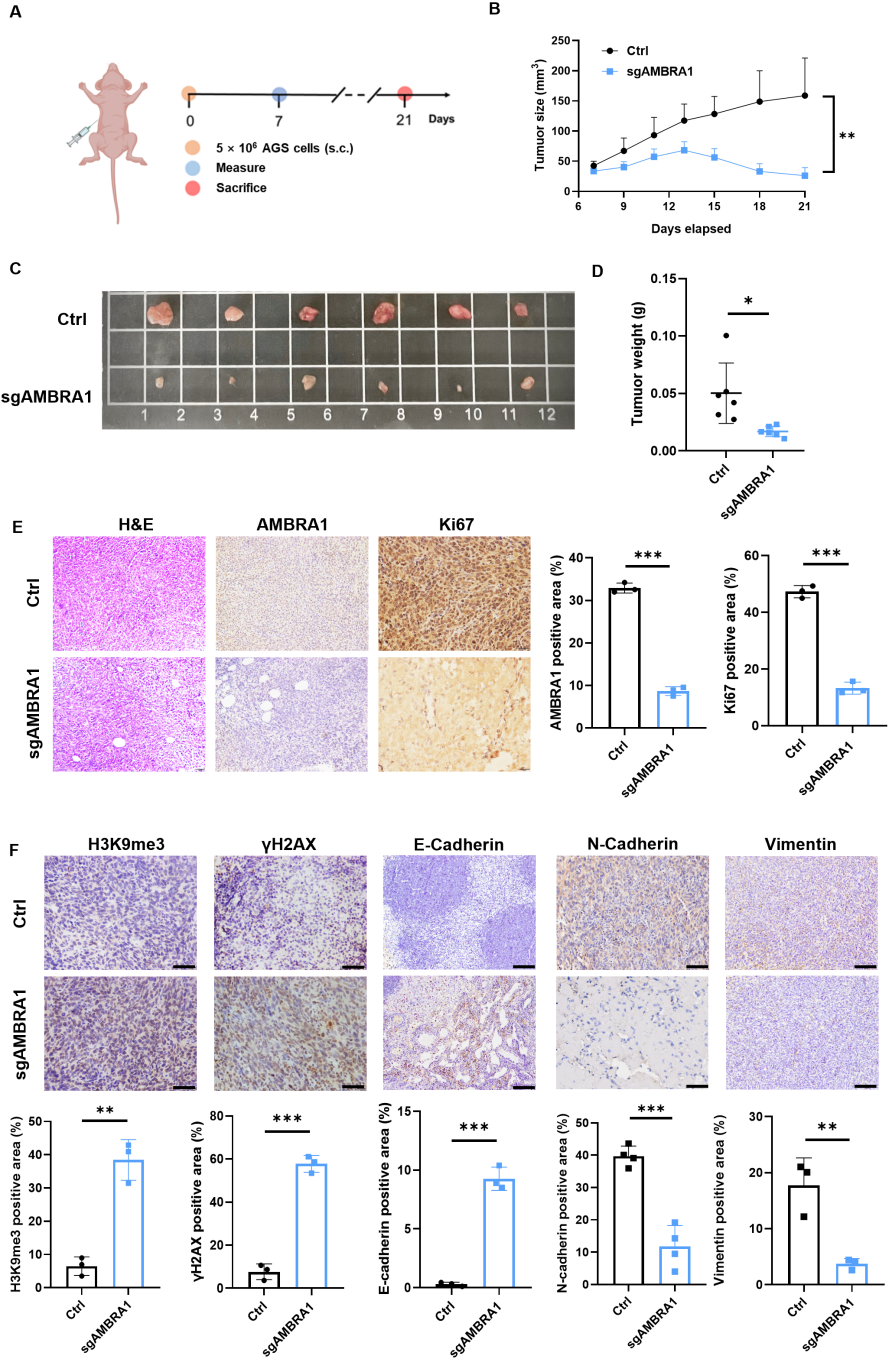
AMBRA1 KO inhibits AGS gastric cancer growth *in vivo*. **(A)** Established a CDX model (n = 6 for each group). **(B)** Growth curves of AGS xenograft tumors in nude mice injected with AGS control cells or AGS cells with CRISPR/Cas9-mediated AMBRA1 KO. Tumor volumes were measured at the indicated time points after injection. **(C)** Representative images of tumors extracted from the control and AMBRA1 KO groups at the experimental endpoint. **(D)** Quantification of tumor weights from the control and AMBRA1 KO groups at the endpoint. **(E, F)** IHC staining and quantification of AMBRA1, Ki-67, H3K9me3 and γH2AX in tumor sections from the control and AMBRA1 KO groups. The scale bar is 50 μm. **(E)** IHC staining and **(F)** quantification of H3K9me3, γH2AX, E-cadherin, N-cadherin and Vimentin in tumor sections from the control and AMBRA1 KO groups. The scale bar is 100 μm. *p < 0.05; **p < 0.01; ***p < 0.001.

To further validate our *in vitro* findings, we conducted *in vivo* experiments using human AGS xenografts. A mixture of 5×10^6^ KO AMBRA1 AGS gastric cancer cells resuspended in sterile PBS and Ceturegel matrix gel into the flank of 6-7 week-old male BALB/c-nude mice, with groups of six mice per experiment. Mice were monitored until the 21-day endpoint, at which point they were sacrificed for further analysis. The result demonstrated that AMBRA1 KO dramatically reduced tumor growth (*p <* 0.01) and size (*p <* 0.05) (**Figures 6A–D**). Histological and immunohistochemical analyses of AMBRA1-KO tumor tissues revealed significant alterations in tumor structure and cellular characteristics compared to control tumors. AMBRA1-KO tumors exhibited markedly disrupted tissue architecture (**Figure 6E**). The tumor cells were loosely organized, lacking the compact structure typically observed in control tumors. Moreover, the structural integrity of the tissue was compromised, with noticeable gaps between cells. A striking feature was the presence of multiple vacuoles throughout the tumor tissue, further indicative of cellular stress or death. Immunohistochemical staining provided additional insights into the cellular state of these tumors. Ki67 staining, a marker of cellular proliferation, was substantially reduced in AMBRA1-depleted tumors, suggesting a significant decrease in the proliferative capacity of these cancer cells. Concurrently, we observed increased expression of senescence-associated markers, specifically H3K9me3 and γH2AX. The upregulation of these markers indicates enhanced cellular senescence and DNA damage in the AMBRA1-KO tumors (**Figures 6E, F**). These histological and immunohistochemical findings strongly corroborate our *in vitro* observations of cell cycle arrest and increased apoptosis in AMBRA1-deficient cells. The loose tissue organization and presence of vacuoles are consistent with increased cell death, while the reduced Ki67 staining aligns with our cell cycle arrest data. The increased senescence markers further support the notion of impaired cellular proliferation and viability in the absence of AMBRA1. The EMT is considered to play a crucial role in the invasive and metastatic behavior of cancer cells. Subsequently, we investigated the impact of AMBRA1 on the expression of EMT-related markers using immunohistochemistry (IHC) in tumor tissues. AMBRA1 significantly enhanced the expression of E-cadherin while simultaneously reducing the levels of N-cadherin and vimentin. Taken together, these results provide compelling *in vivo* evidence for the critical role of AMBRA1 in maintaining tumor growth and cellular integrity in gastric cancer.

These findings revealed the multifaceted role of AMBRA1 in regulating cellular senescence in gastric cancer cells. The observed increases in DDR markers, and senescence-associated histone modifications in AMBRA1-deficient cells highlight the protein’s importance in maintaining cellular homeostasis. Furthermore, the enrichment analysis of senescence-related gene sets in patient samples provides a broader context for understanding the relevance of these cellular processes in gastric cancer progression and potential therapeutic interventions. And our data suggest that increased ROS levels resulting from siAMBRA1 exposure activate the DDB1/FOXO3/CDKN1/CCND1 signaling pathway. This activated pathway appears to be involved in the anti-proliferative effect of siAMBRA1 by inducing senescence in AGS cells.

## 4 Discussion

STAD remains a significant clinical challenge due to limited treatment efficacy and incomplete understanding of its molecular drivers (29). The E3 ligase adaptor, AMBRA1 regulates autophagy and plays a crucial role in tumor initiation and development. Furthermore, it contributes to preserving the integrity of the intracellular environment (8). A growing number of studies have reported that AMBRA1 functions as a tumor suppressor in melanoma (9, 13). In contrast to its tumor suppressor role in melanoma, our findings revealed that AMBRA1 functions as an oncogene in STAD. This duality may be influenced by various factors, including the specific tumor microenvironment, signaling pathways activated in different contexts, and the cellular heterogeneity within tumors. Our study revealed that AMBRA1 is highly expressed in STAD tissues compared to normal tissues, and this overexpression correlates with poor patient prognosis (**Figure 1**). *In vitro* and *in vivo* experiments demonstrated that AMBRA1 depletion significantly impairs STAD cell growth, migration, and invasion (**Figure 2**; **Supplementary Figure S4**). The invasiveness and metastasis of gastric cancer are known to be significantly influenced by EMT (30). The EMT process has a positive correlation with increased invasion and migration. Prior studies have demonstrated that induced expression of E-cadherin can impede the migration and metastasis of gastric cancer cells, while E-cadherin deletion enhances these processes (30). In gastric cancer, we found that AMBRA1-KO dramatically reduced the levels of vimentin and N-cadherin and increased the expression of E-cadherin. Furthermore, there was a noticeable decrease in the migration and invasion of gastric cancer cells in AMBRA1-KO. These results imply that AMBRA1-KO limits gastric cancer cells’ ability to migrate and invade by inhibiting the EMT process. These findings revealed that AMBRA1 appears to play a critical role in regulating the plasticity of gastric cancer cells, particularly in terms of proliferation, migration, invasion, cellular senescence and EMT.

Moreover, our findings indicate that high AMBRA1 expression correlates with poor overall survival in STAD patients and is positively associated with T cell CD4+ infiltration (**Supplementary Figure S1**). This suggests that AMBRA1 may play a crucial role in modulating immune responses, potentially affecting the tumor’s ability to evade immune surveillance. The relationship between AMBRA1 expression and immune cell infiltration, particularly T cells, underscores the importance of understanding how AMBRA1 may influence the immune microenvironment in gastric cancer. Moreover, we observed that mutations in AMBRA1 could lead to a significant reduction in T cell CD4+ memory cell infiltration, which may weaken the immune response against tumors. This highlights the potential for AMBRA1 to serve as a biomarker for predicting immune response and treatment outcomes in STAD. Future research should focus on elucidating AMBRA1’s influence on various immune cell types, such as T cells and macrophages, and their roles in tumor progression or suppression within the STAD.

The development of cellular senescence in AMBRA1-deficient STAD cells is one of our study’s most exciting discoveries; it reveals a novel mechanism by which AMBRA1 promotes tumor progression. A persistent condition of cell cycle stoppage known as cellular senescence is thought to be a tumor-suppressive mechanism that may be used in cancer treatment. It comprises early senescence brought on by a variety of stresses and replicative senescence brought on by telomere shortening (31). Telomere shortening or malfunction triggers a typical DNA-damage response that leads to replicative senescence (32). However, a number of external stresses, including oxidative stress, DNA damage, and the activation of certain oncogenes, are responsible for premature senescence (31). In this study, we were the first to demonstrate AMBRA1’s crucial responsibility in managing cellular senescence in STAD. The increased proportion of SA-β-Gal positive cells, elevated ROS levels, and shortened telomeres in AMBRA1 KD cells provide strong evidence for the activation of senescence programs (**Figure 4**). This senescence induction is further supported by the upregulation of DDR markers (γ-H2AX and H3K9me3) and the activation of the DDB1/FOXO3/CDKN1/CCND1 signaling pathway (**Figures 5**, **6**). These results suggest that AMBRA1 plays a crucial role in preventing replicative and premature senescence in STAD, possibly contributing to their sustained proliferative capacity.

Building on these findings of cellular senescence, we further investigated AMBRA1’s role in cell cycle regulation and apoptosis. Importantly, cell senescence is considered a natural tumor-suppressive mechanism, and cell apoptosis can be viewed as the ultimate stage of cell senescence. Recent studies showed that AMBRA1 is a crucial regulator of Cyclin D1 stability, which is essential for the advancement of the cell cycle (9–11). The cell cycle arrest and increased apoptosis observed in AMBRA1-deficient cells further highlight its importance in maintaining cancer cell viability. The decrease in cyclin D1 levels and G1 phase arrest indicate that AMBRA1 is essential for cell cycle progression. Moreover, the significant increase in apoptotic cells upon AMBRA1 silencing suggests that it may function as an anti-apoptotic factor in STAD cells (**Figure 3**). This perspective underscores the significance of our findings regarding AMBRA1’s role in regulating both senescence and apoptosis in STAD cells.

**Figure 7:**
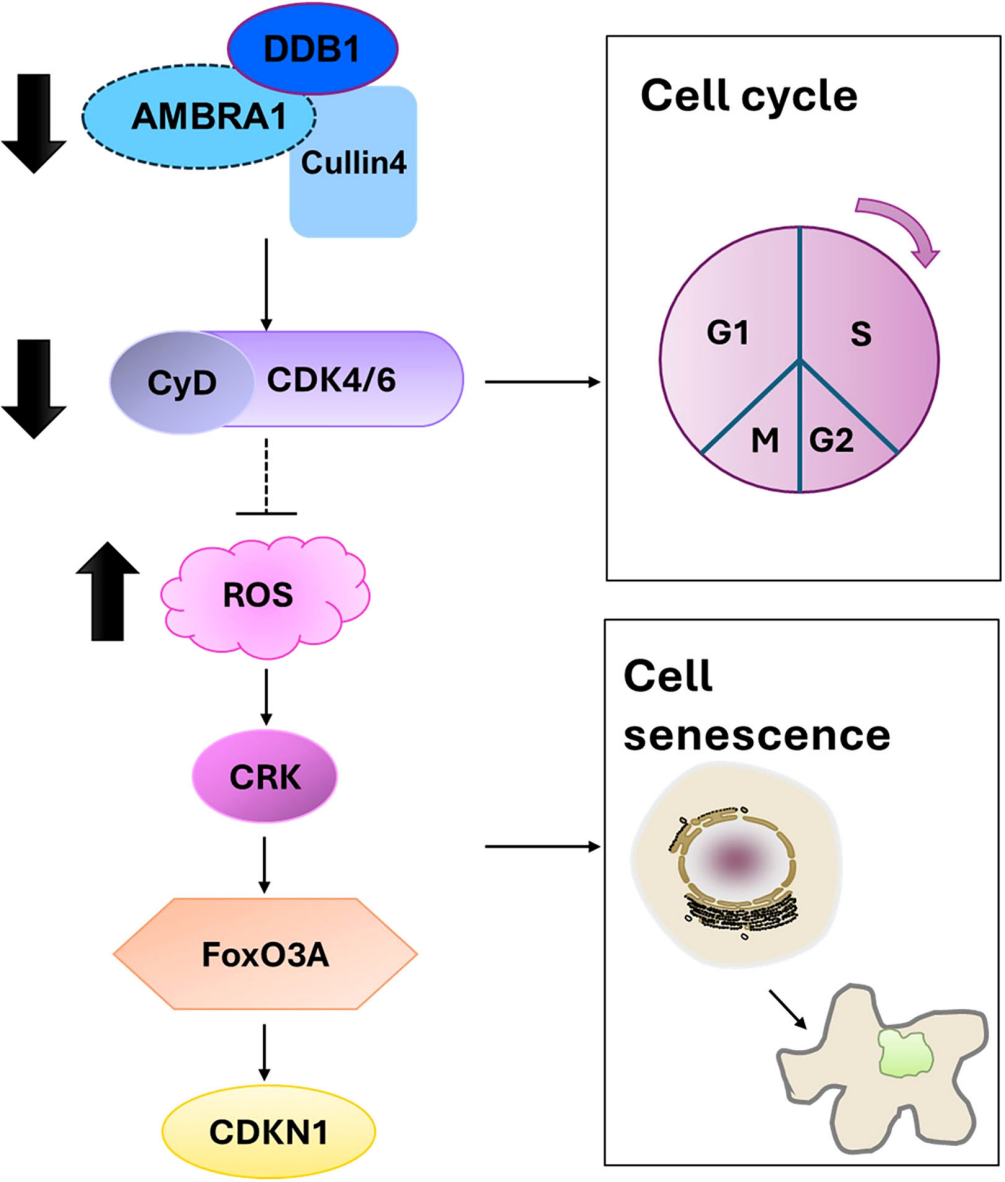
Proposed mechanism by which AMBRA1 regulates gastric cancer cell proliferation and tumorigenesis.

Beyond its effects on individual cellular processes, our findings on AMBRA1’s role in cellular senescence and apoptosis provide crucial insights into its broader function in regulating tumor plasticity in STAD. Our findings on AMBRA1’s role in cellular senescence and apoptosis provide crucial insights into its broader function in regulating tumor plasticity in STAD. Tumor plasticity refers to the ability of cancer cells to adapt their phenotype in response to various stressors, including therapeutic interventions. The observed effects of AMBRA1 deficiency on cell cycle progression, senescence induction, and apoptosis directly impact this plasticity. By regulating these fundamental cellular processes, AMBRA1 appears to maintain a more flexible, adaptable state in STAD cells. When AMBRA1 is depleted, cells lose this plasticity, becoming more susceptible to senescence and apoptosis. This loss of plasticity is evident in the reduced proliferation, migration, and invasion capabilities of AMBRA1-deficient cells, as well as their increased sensitivity to various stressors and chemotherapeutic agents. Furthermore, the epigenetic changes observed, particularly in H3K9me3 levels, suggest that AMBRA1 may influence plasticity through chromatin remodeling, potentially affecting the expression of genes involved in cell fate decisions. Thus, AMBRA1 emerges as a key regulator of tumor plasticity in STAD, orchestrating a delicate balance between proliferation, senescence, and cell death (**Figure 7**).

## 5 Conclusion

In conclusion, our study reveals AMBRA1 as a critical oncogenic driver in STAD, regulating tumor plasticity, proliferation, and resistance to cellular senescence and apoptosis. Through *in vitro* and *in vivo* studies, we demonstrated that AMBRA1 depletion significantly impairs tumor growth, alters cellular architecture, and increases markers of senescence and DNA damage. These findings highlight AMBRA1’s multifaceted role in STAD progression and its potential as both a prognostic biomarker.

## Data Availability

The raw data supporting the conclusions of this article will be made available by the authors, without undue reservation.
