# Supplementary material for: AMBRA1 drives gastric cancer progression through regulation of tumor plasticity
Source: Front Immunol. 2024 Dec 10;15:1494364. doi: 10.3389/fimmu.2024.1494364 (PMC11666514; doi:10.3389/fimmu.2024.1494364)
Supplement: Supplementary file 1 [file DataSheet1.pdf]

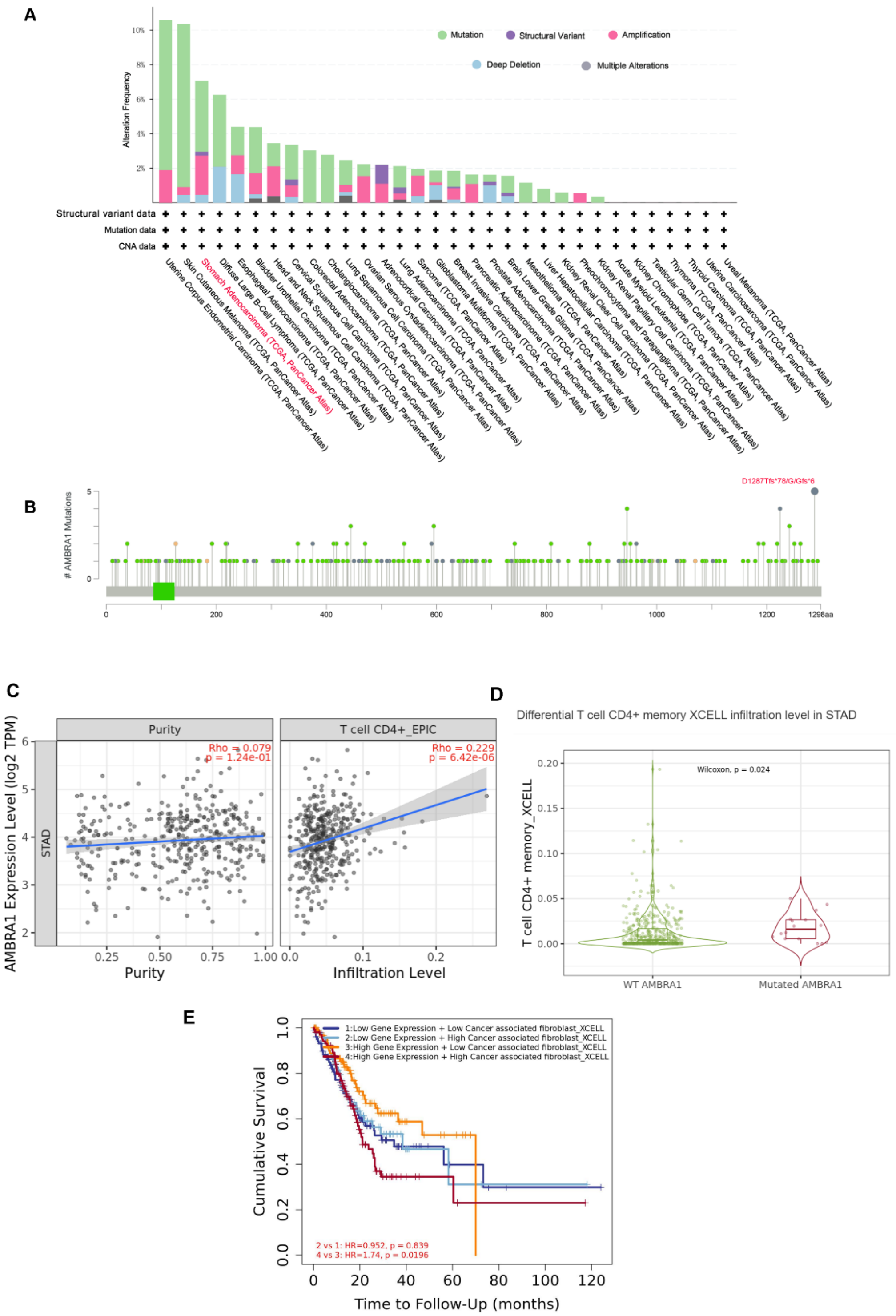

Figure S1 (A) AMBRA1 mutation characteristics in various TCGA cancers using the cBioPortal tool. (B) The frequency of change and kind of mutation and mutation site. AMBRA1 alteration frequencies in various cancers and mutation sites were visualized. (C) Correlation between AMBRA1 expression (log2 TPM) and tumor purity/T cell CD4+ infiltration levels in STAD. (D) Comparison of T cell CD4+ memory infiltration levels in STAD with wild-type (WT) and mutated AMBRA1. (E) Kaplan-Meier survival curve showing cumulative survival over time in relation to AMBRA1 expression levels in STAD patients.

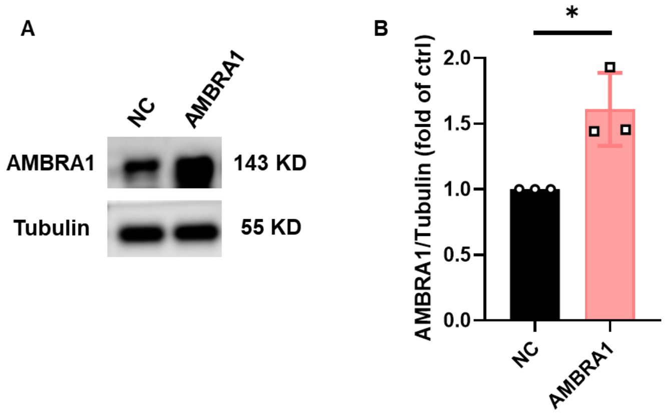

Figure S2 (A) Western blotting analysis of the AMBRA1 levels after the treatment of control plasmid and AMRBA1 overexpression plasmid. (B) Quantitative results of the AMBRA1 levels in AGS cells following transfection of AMBRA1 overexpression plasmid. *$p < 0.05$; **$p < 0.01$; ***$p < 0.001$; ****$p < 0.0001$

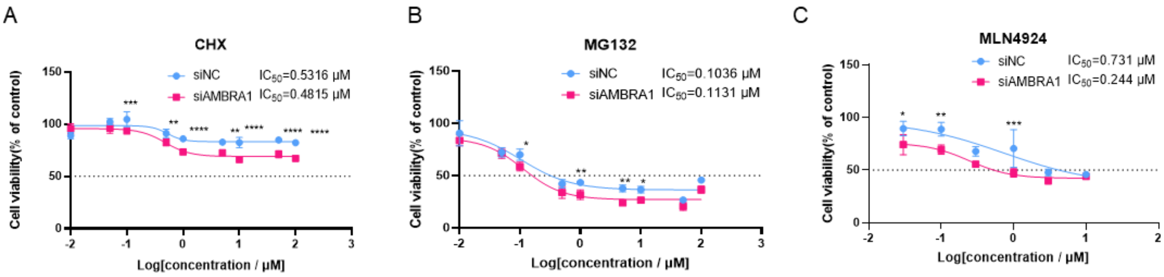

Figure S3 (A) CHX inhibits the growth of AGS cells and KD-AMBRA1 AGS cells. The IC_{50} values were determined after 24 hours of treatment with CHX. (B) MG132 inhibits the growth of AGS cells and KD-AMBRA1 AGS cells. The IC_{50} values were determined after 24 hours of treatment with MG132. (C) MLN4924 inhibits the growth of AGS cells and KD-AMBRA1 AGS cells. The IC_{50} values were determined after 24 hours of treatment with MLN4924.

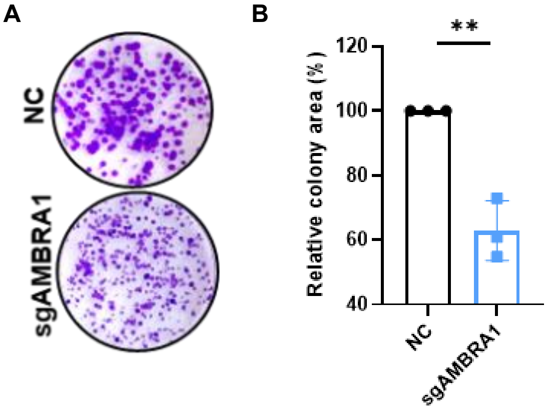

Figure S4 (A) Representative images and (B) quantification of relative colony area of ctrl group and AMBRA1 sgRNA group stained after 14 days (n = 3 for each group).

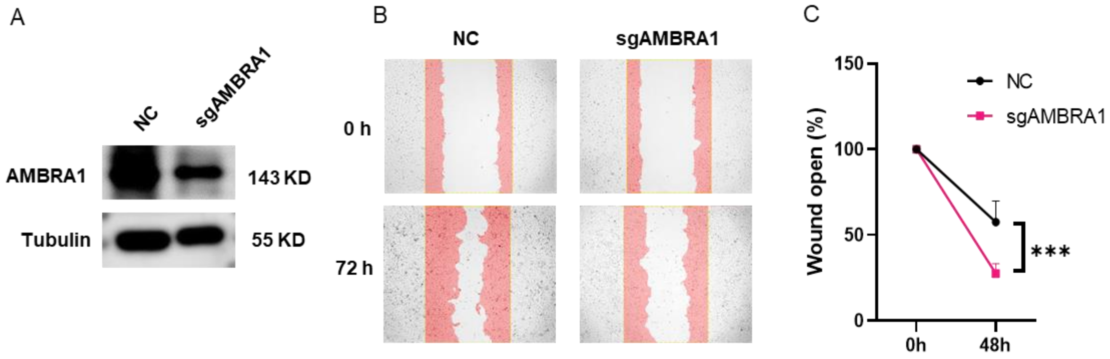

Figure S5 (A) Western blotting analysis of the AMBRA1 levels after the treatment of control and AMBRA1 sgRNA. (B) Representative images and (C) quantitative results for the Wound healing for AGS cells with AMBRA1 sgRNA transfection. *$p < 0.05$; **$p < 0.01$; ***$p < 0.001$; ****$p < 0.0001$

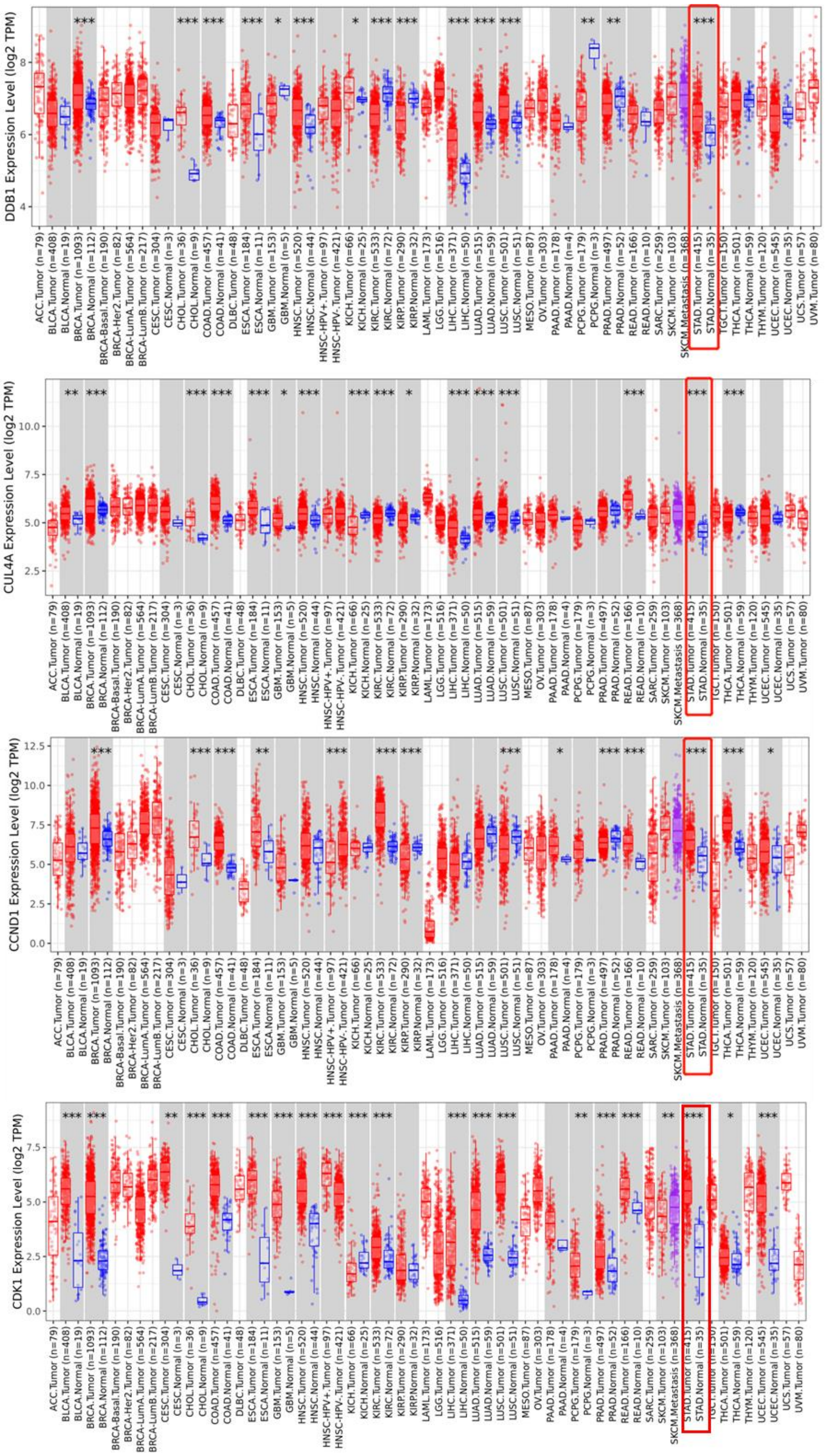

Figure S6 The samples' log2 (TPM) values for DDB1, CUL4A, CCND1(cyclin D1) and CDK1(CDKN1) expression are shown on the Y-axis. This study investigates AMBRA1 pan-cancer expression in tumor and normal tissues using data from the TCGA database and the TIMER tool. *$p < 0.05$; **$p < 0.01$; ***$p < 0.001$; ****$p < 0.0001$
