# Supplementary material for: AMBRA1 drives gastric cancer progression through regulation of tumor plasticity
Source: Front Immunol. 2024 Dec 10;15:1494364. doi: 10.3389/fimmu.2024.1494364 (PMC11666514; doi:10.3389/fimmu.2024.1494364)
Supplement: Supplementary file 2 [file DataSheet2.zip › AMBRA1-Western Blot source data/Source Data suppl final.pptx]

## Slide 1
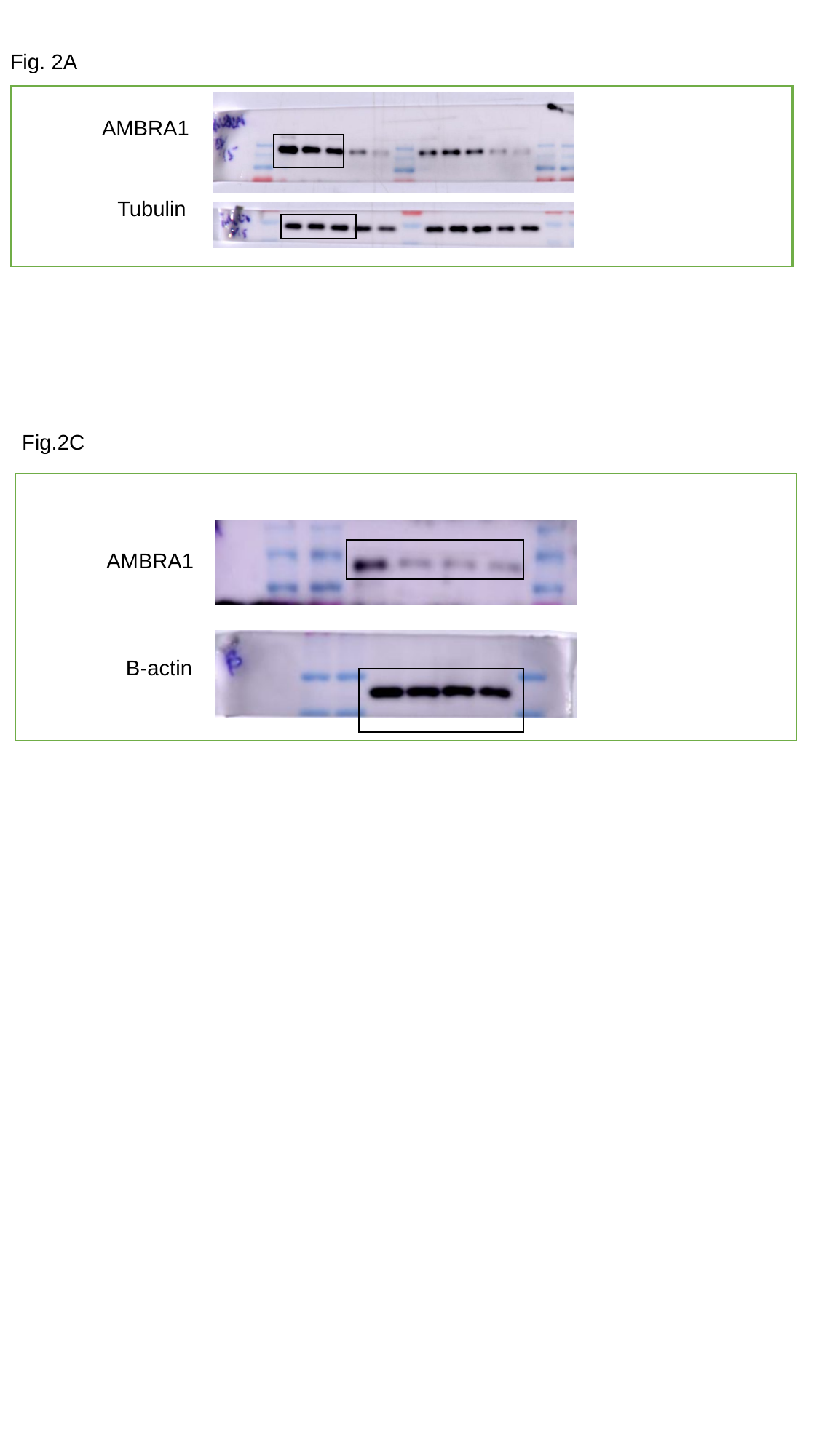

Fig. 2A
AMBRA1
Tubulin
Fig.2C
AMBRA1
Β-actin

## Slide 2
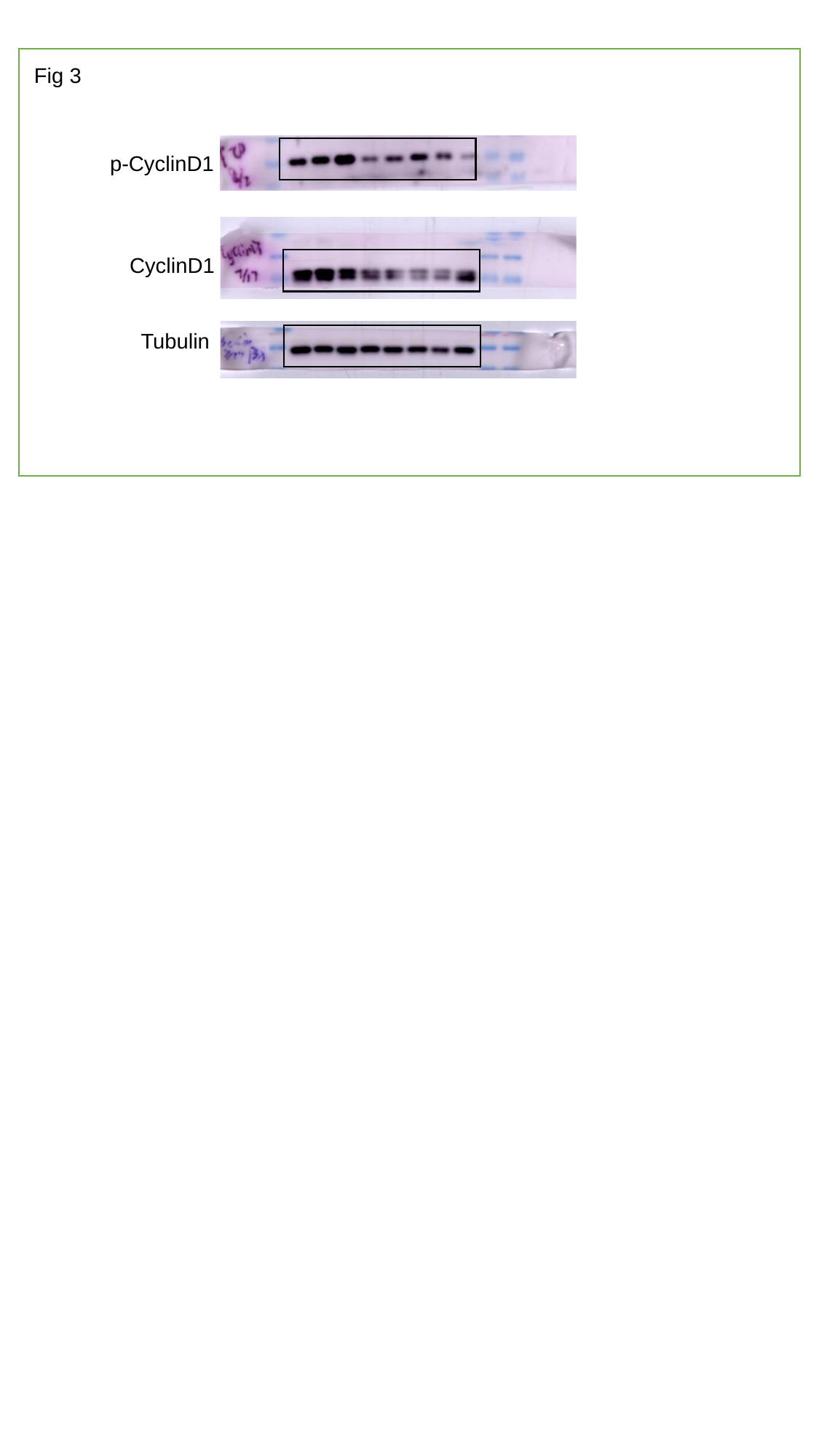

Fig 3
p-CyclinD1
CyclinD1
Tubulin

## Slide 3
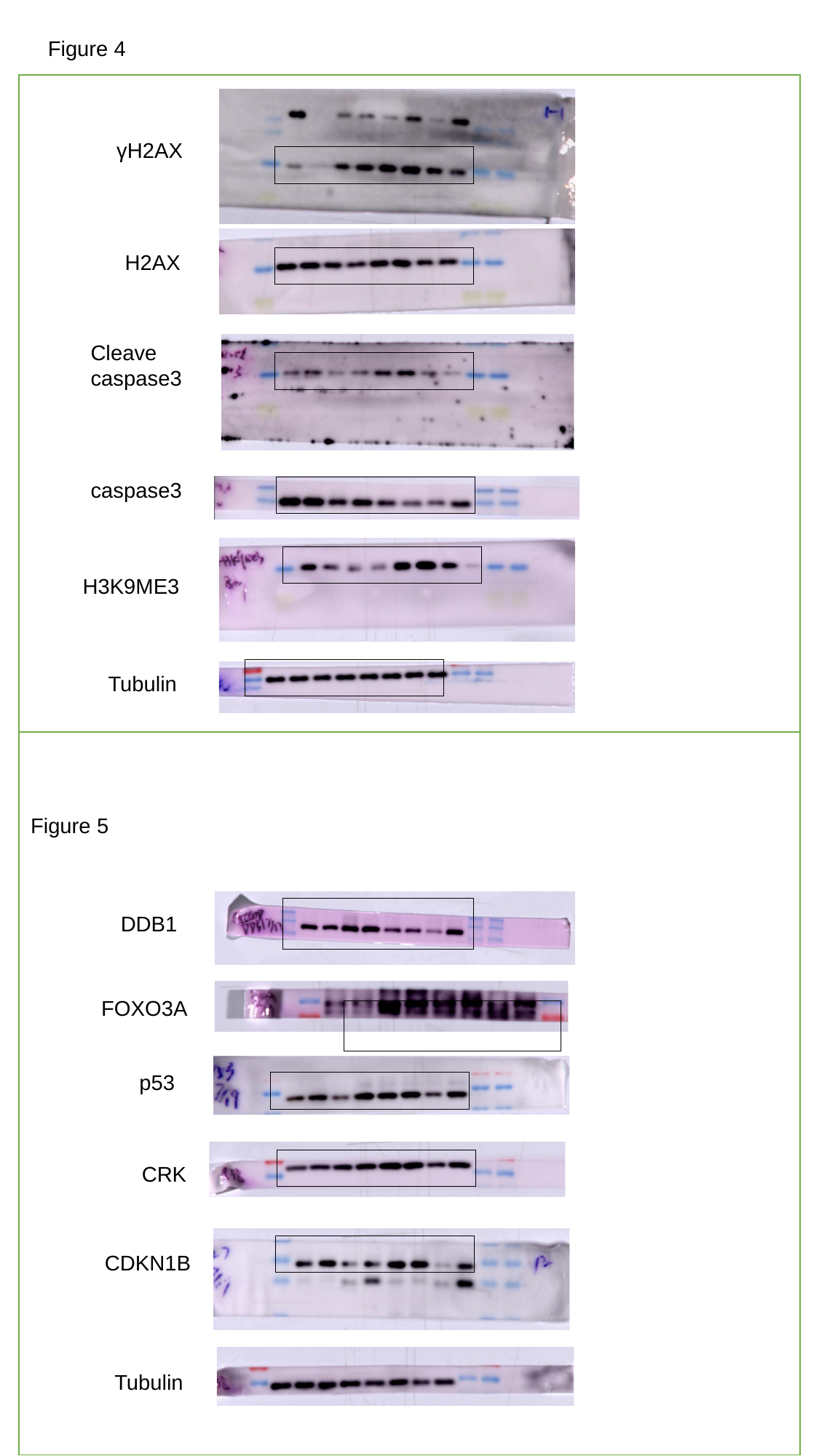

Figure 4
γH2AX
H2AX
Cleave caspase3
caspase3
H3K9ME3
Tubulin
Figure 5
DDB1
FOXO3A
p53
CRK
CDKN1B
Tubulin

## Slide 4
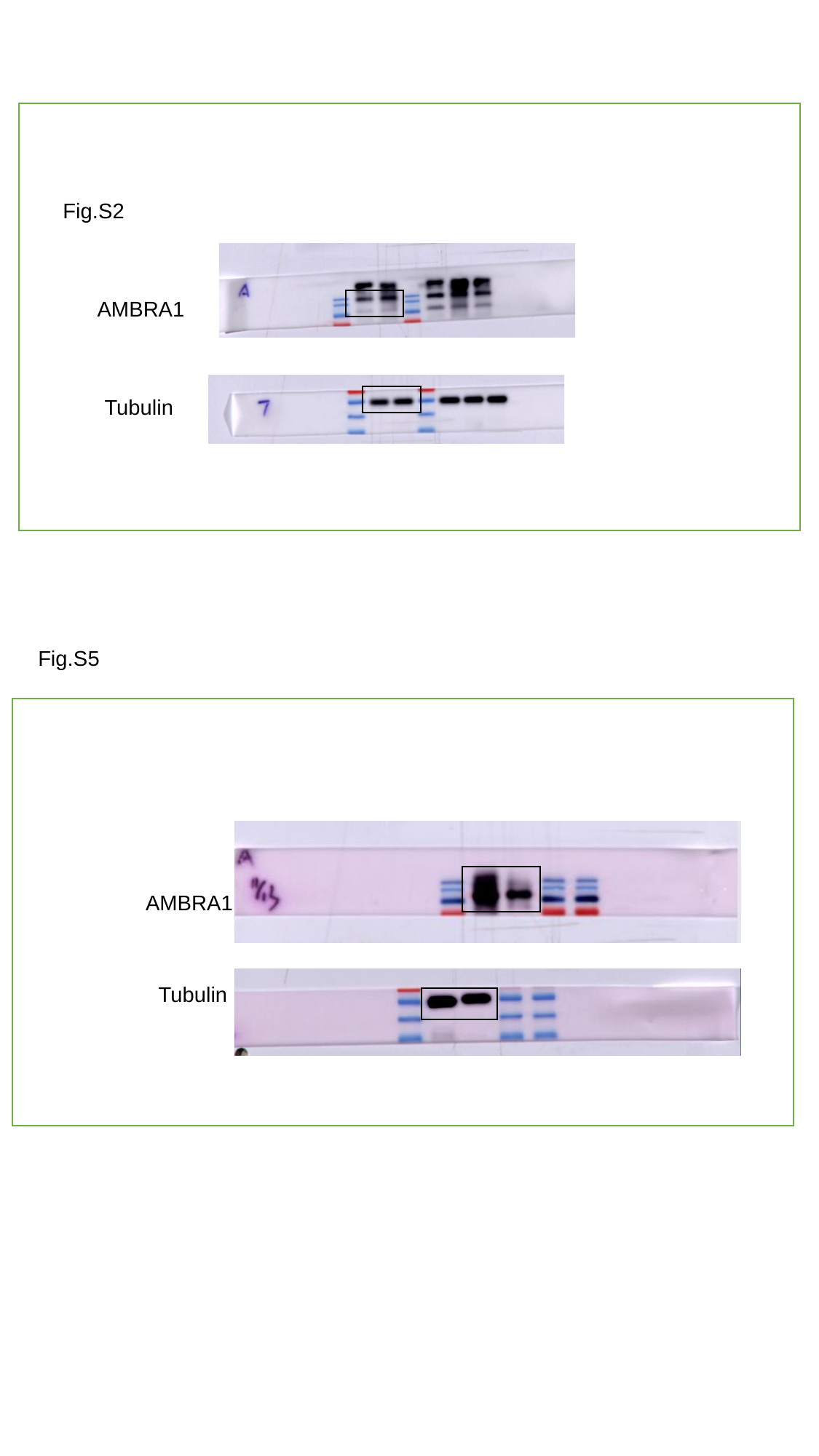

Fig.S2
AMBRA1
Tubulin
Fig.S5
AMBRA1
Tubulin
