# Supplementary figures and images for: AMBRA1 drives gastric cancer progression through regulation of tumor plasticity
Source: Front Immunol. 2024 Dec 10;15:1494364. doi: 10.3389/fimmu.2024.1494364 (PMC11666514; doi:10.3389/fimmu.2024.1494364)

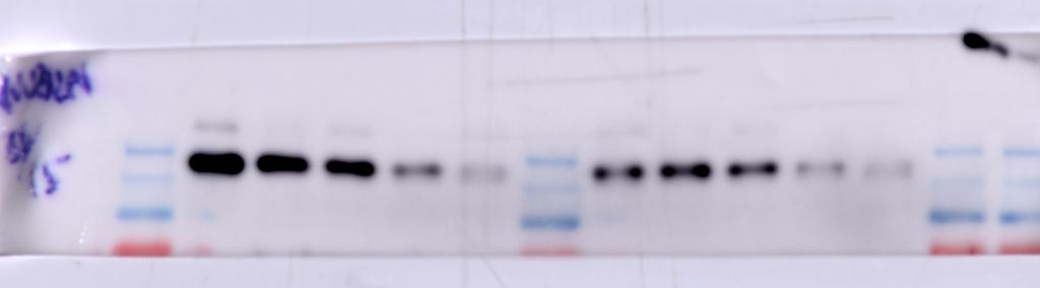

Supplement: Supplementary file 2 [file DataSheet2.zip › AMBRA1-Western Blot source data/Fig.2/Fig.2A/AMBRA1.tif]

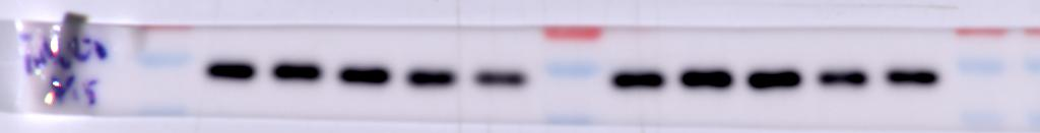

Supplement: Supplementary file 2 [file DataSheet2.zip › AMBRA1-Western Blot source data/Fig.2/Fig.2A/tubulin.tif]

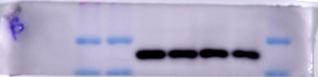

Supplement: Supplementary file 2 [file DataSheet2.zip › AMBRA1-Western Blot source data/Fig.2/Fig.2C/actin.tif]

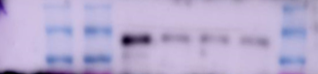

Supplement: Supplementary file 2 [file DataSheet2.zip › AMBRA1-Western Blot source data/Fig.2/Fig.2C/AMBRA1-siRNA.tif]

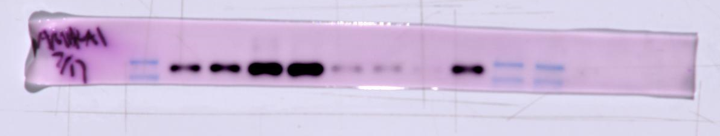

Supplement: Supplementary file 2 [file DataSheet2.zip › AMBRA1-Western Blot source data/Fig.3/AMBRA1.tif]

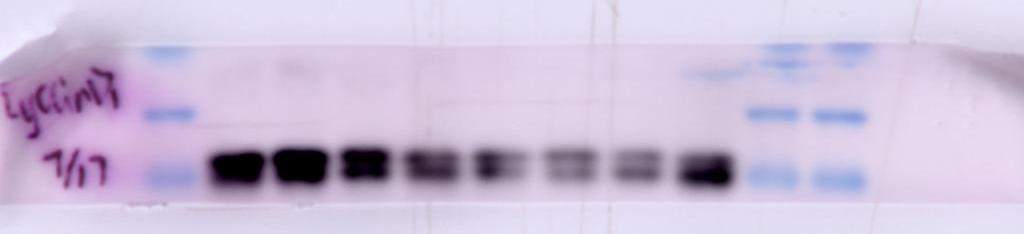

Supplement: Supplementary file 2 [file DataSheet2.zip › AMBRA1-Western Blot source data/Fig.3/cyclind1.tif]

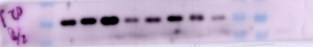

Supplement: Supplementary file 2 [file DataSheet2.zip › AMBRA1-Western Blot source data/Fig.3/pcyclind1.tif]

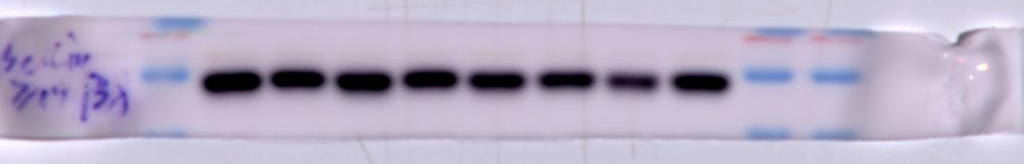

Supplement: Supplementary file 2 [file DataSheet2.zip › AMBRA1-Western Blot source data/Fig.3/Tubulin.tif]

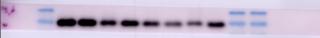

Supplement: Supplementary file 2 [file DataSheet2.zip › AMBRA1-Western Blot source data/Fig.4/caspase3.tif]

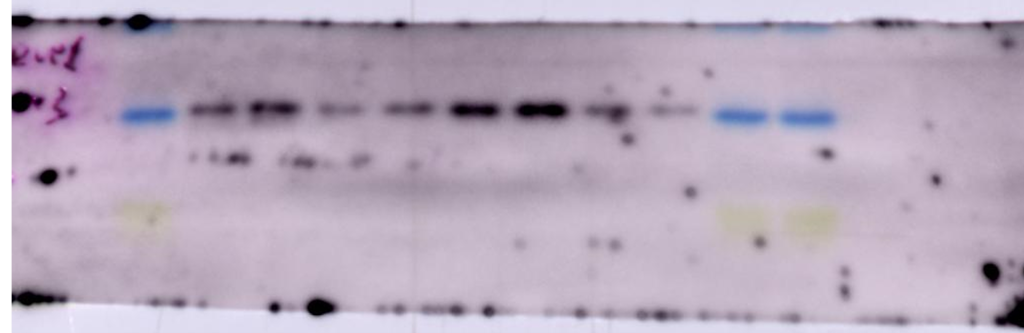

Supplement: Supplementary file 2 [file DataSheet2.zip › AMBRA1-Western Blot source data/Fig.4/Cleave caspase3.tif]

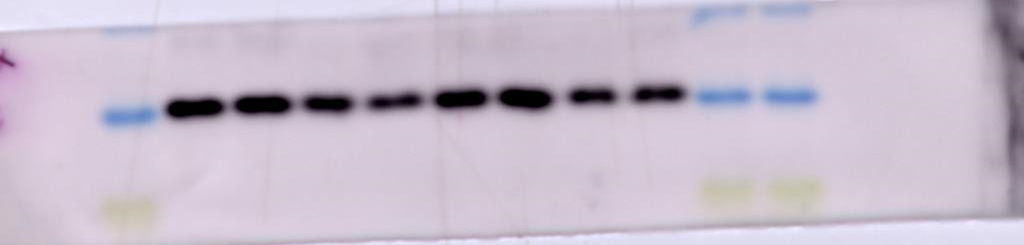

Supplement: Supplementary file 2 [file DataSheet2.zip › AMBRA1-Western Blot source data/Fig.4/H2AX.tif]

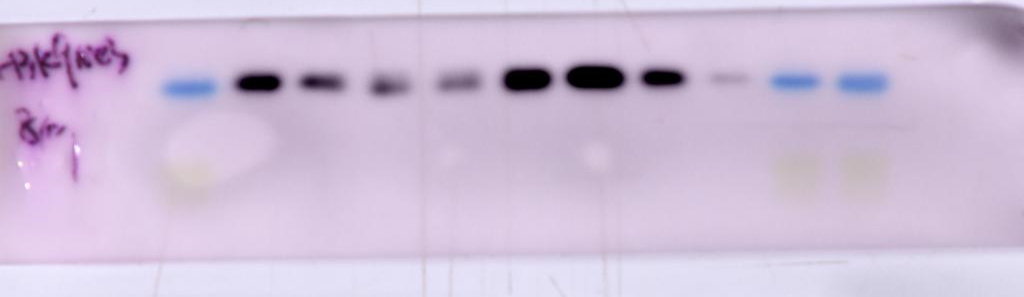

Supplement: Supplementary file 2 [file DataSheet2.zip › AMBRA1-Western Blot source data/Fig.4/H3K9ME3.jpg.tif]

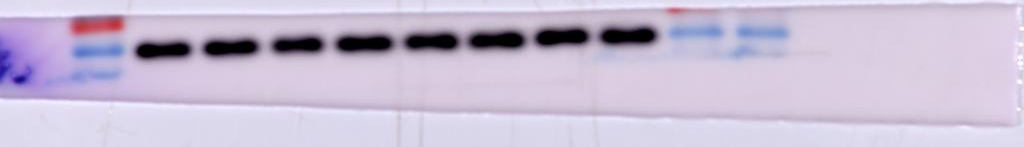

Supplement: Supplementary file 2 [file DataSheet2.zip › AMBRA1-Western Blot source data/Fig.4/tubulin.jpg.tif]

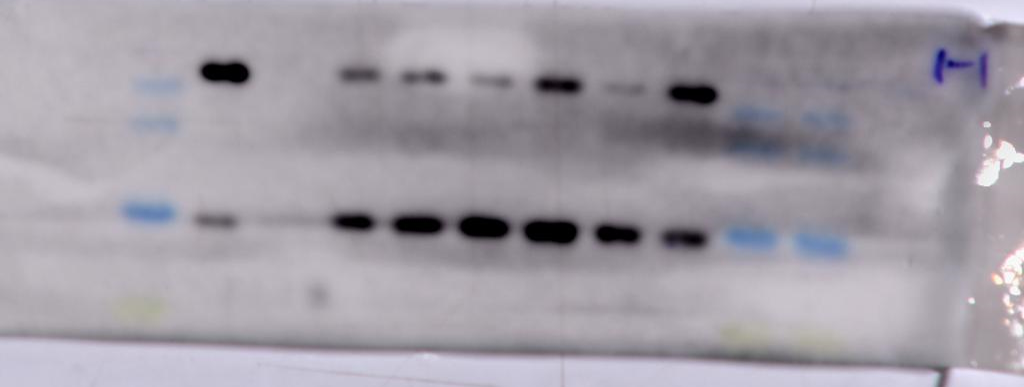

Supplement: Supplementary file 2 [file DataSheet2.zip › AMBRA1-Western Blot source data/Fig.4/γH2AX.tif]

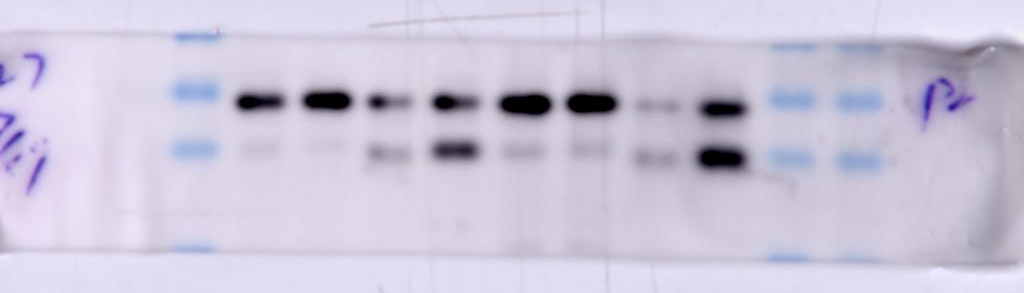

Supplement: Supplementary file 2 [file DataSheet2.zip › AMBRA1-Western Blot source data/Fig.5/CDKN1B.tif]

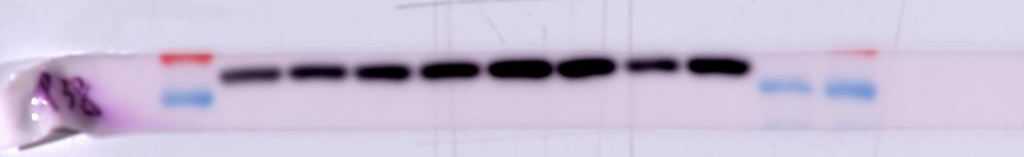

Supplement: Supplementary file 2 [file DataSheet2.zip › AMBRA1-Western Blot source data/Fig.5/CRK.tif]

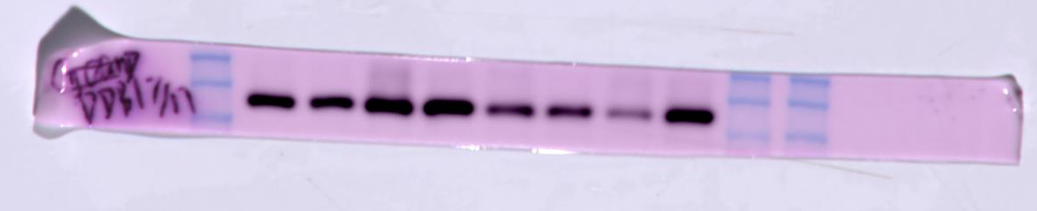

Supplement: Supplementary file 2 [file DataSheet2.zip › AMBRA1-Western Blot source data/Fig.5/DDB1.tif]

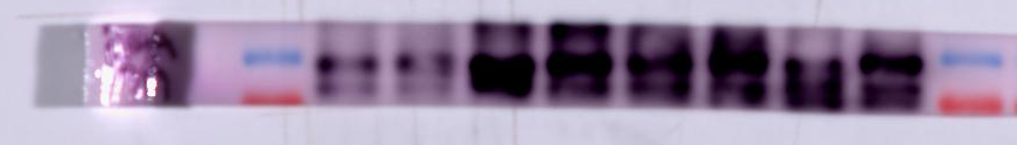

Supplement: Supplementary file 2 [file DataSheet2.zip › AMBRA1-Western Blot source data/Fig.5/FOXO3A.tif]

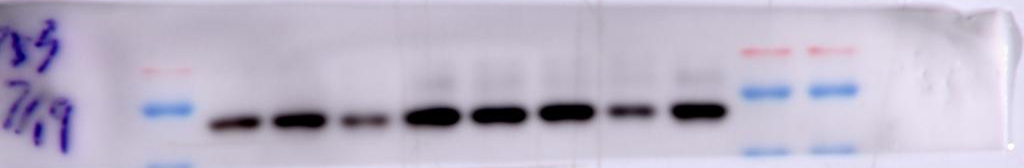

Supplement: Supplementary file 2 [file DataSheet2.zip › AMBRA1-Western Blot source data/Fig.5/p53.tif]

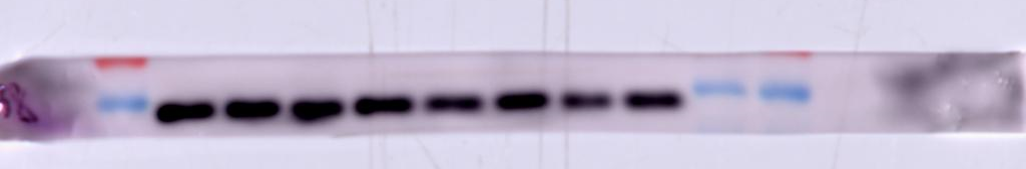

Supplement: Supplementary file 2 [file DataSheet2.zip › AMBRA1-Western Blot source data/Fig.5/TUBULIN.tif]

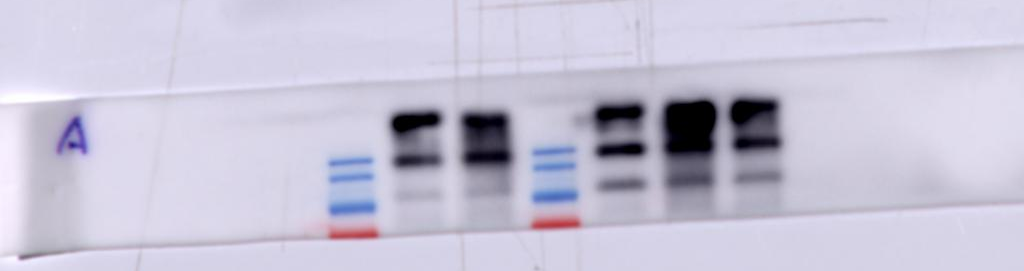

Supplement: Supplementary file 2 [file DataSheet2.zip › AMBRA1-Western Blot source data/Fig.S2/AMBRA1.tif]

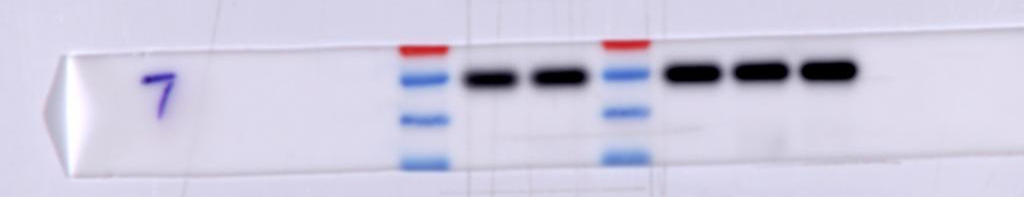

Supplement: Supplementary file 2 [file DataSheet2.zip › AMBRA1-Western Blot source data/Fig.S2/TUBULIN.tif]

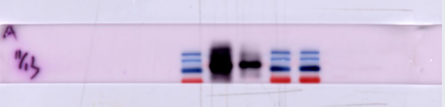

Supplement: Supplementary file 2 [file DataSheet2.zip › AMBRA1-Western Blot source data/Fig.S5/AMBRA1.tif]

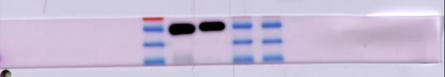

Supplement: Supplementary file 2 [file DataSheet2.zip › AMBRA1-Western Blot source data/Fig.S5/TUBULIN.tif]
